# A Pragmatic Cluster Randomized Trial of an Electronic Clinical Decision Support System to Improve Chronic Kidney Disease Management in Primary Care: Design, Rationale, and Implementation Experience

**DOI:** 10.2196/14022

**Published:** 2019-06-07

**Authors:** Elaine C Khoong, Leah Karliner, Lowell Lo, Marilyn Stebbins, Andrew Robinson, Sarita Pathak, Jasmine Santoyo-Olsson, Rebecca Scherzer, Carmen A Peralta

**Affiliations:** 1 Division of General Internal Medicine at Zuckerberg San Francisco General Hospital Department of Medicine University of California San Francisco San Francisco, CA United States; 2 Division of General Internal Medicine Department of Medicine University of California San Francisco San Francisco, CA United States; 3 Multiethnic Health Equity Research Center University of California San Francisco San Francisco, CA United States; 4 Division of Nephrology Department of Medicine University of California San Francisco San Francisco, CA United States; 5 School of Pharmacy University of California San Francisco San Francisco, CA United States; 6 University of California San Francisco San Francisco, CA United States; 7 Kidney Health Research Collaborative San Francisco Veterans Affairs Medical Center University of California San Francisco San Francisco, CA United States; 8 Cricket Health San Francisco, CA United States

**Keywords:** chronic kidney disease, clinical decision support systems, pragmatic clinical trial, electronic health records

## Abstract

**Background:**

The diagnosis of chronic kidney disease (CKD) is based on laboratory results easily extracted from electronic health records; therefore, CKD identification and management is an ideal area for targeted electronic decision support efforts. Early CKD management frequently occurs in primary care settings where primary care providers (PCPs) may not implement all the best practices to prevent CKD-related complications. Few previous studies have employed randomized trials to assess a CKD electronic clinical decision support system (eCDSS) that provided recommendations to PCPs tailored to each patient based on laboratory results.

**Objective:**

The aim of this study was to report the trial design and implementation experience of a CKD eCDSS in primary care.

**Methods:**

This was a 3-arm pragmatic cluster-randomized trial at an academic general internal medicine practice. Eligible patients had 2 previous estimated-glomerular-filtration-rates by serum creatinine (eGFR_Cr_) <60 mL/min/1.73m^2^ at least 90 days apart. Randomization occurred at the PCP level. For patients of PCPs in either of the 2 intervention arms, the research team ordered triple-marker testing (serum creatinine, serum cystatin-c, and urine albumin-creatinine-ratio) at the beginning of the study period, to be completed when acquiring labs for regular clinical care. The eCDSS launched for PCPs and patients in the intervention arms during a regular PCP visit subsequent to completing the triple-marker testing. The eCDSS delivered individualized guidance on cardiovascular risk-reduction, potassium and proteinuria management, and patient education. Patients in the eCDSS+ arm also received a pharmacist phone call to reinforce CKD-related education. The primary clinical outcome is blood pressure change from baseline at 6 months after the end of the trial, and the main secondary outcome is provider awareness of CKD diagnosis. We also collected process, patient-centered, and implementation outcomes.

**Results:**

A multidisciplinary team (primary care internist, nephrologists, pharmacist, and informaticist) designed the eCDSS to integrate into the current clinical workflow. All 81 PCPs contacted agreed to participate and were randomized. Of 995 patients initially eligible by eGFR_Cr_, 413 were excluded per protocol and 58 opted out or withdrew, resulting in 524 patient participants (188 usual care; 165 eCDSS; and 171 eCDSS+). During the 12-month intervention period, 53.0% (178/336) of intervention patient participants completed triple-marker labs. Among these, 138/178 (77.5%) had a PCP appointment after the triple-marker labs resulted; the eCDSS was opened for 73.9% (102/138), with orders or education signed for 81.4% (83/102).

**Conclusions:**

Successful integration of an eCDSS into primary care workflows and high eCDSS utilization rates at eligible visits suggest this tailored electronic approach is feasible and has the potential to improve guideline-concordant CKD care.

**Trial Registration:**

ClinicalTrials.gov NCT02925962; https://clinicaltrials.gov/ct2/show/NCT02925962 (Archived by WebCite at http://www.webcitation.org/78qpx1mjR)

**International Registered Report Identifier (IRRID):**

DERR1-10.2196/14022

## Introduction

### Management of Early Chronic Kidney Disease

Chronic kidney disease (CKD) is common in adults; in the United States, 14% of all adults and nearly half of individuals aged ≥70 years have CKD [1]. CKD is an important predictor of morbidity and cardiovascular mortality [2]. Although most patients with early CKD are seen by primary care providers (PCPs), studies have consistently shown that patients and PCPs remain largely unaware of the patient’s CKD diagnosis until the disease is more advanced [3,4]. Even when early CKD is recognized, PCPs frequently are unaware of the best practices for risk stratification and prevention of CKD-related complications [3]. In particular, both CKD staging and complication risk stratification are greatly improved using a triple marker strategy: urine albumin-creatinine ratio (ACR), estimated glomerular filtration rate (eGFR) based on both serum creatinine levels (eGFR_Cr_) and cystatin-c levels (eGFR_Cys_) [5-9]. Early detection of CKD and risk stratification enable clinicians and patients to take individualized actions that improve outcomes and have the potential to attenuate progression, such as blood pressure management and renin-angiotensin blockade [10-13], statin therapy [13-16], avoidance of nephrotoxic medications [17,18], and glucose management in individuals with diabetes [19-21].

### Approaches to Increase Guideline-Concordant Early Chronic Kidney Disease Management

With the adoption of electronic health records (EHRs), there have been opportunities to implement low-cost interventions to improve patient care. Given that CKD diagnosis is based on laboratory results easily extracted from EHRs, CKD identification and management is an ideal area for targeted electronic efforts [22,23]. Previous studies have shown the feasibility of electronic clinical decision support systems (eCDSS) to improve guideline-concordant care for patients with CKD [24-29]. However, most studies have not been randomized nor have they provided individualized recommendations for patients based on laboratory results. Instead, they have utilized standard reminders or checklists. In addition, concerns about the eCDSS being burdensome or disruptive to workflow have hindered the development and testing of more complex, individualized eCDSS [23,30,31].

Team-based approaches for providing chronic disease management in primary care practice have also been shown to improve patient outcomes [32,33]. For example, blood pressure (BP) control is improved when managed by nonphysician members of the team, including nurses and pharmacists [34-37]. Initial studies have suggested that CKD, like other chronic diseases, may also benefit from a team-based approach [38,39].

### Trial Aims

Given the promise of both eCDSS and team-based care to improve guideline-concordant CKD care in primary care practice, we designed a pragmatic cluster randomized 3-arm trial (usual care; eCDSS; and eCDSS plus pharmacist follow-up). This trial aimed to assess the feasibility and impact of a CKD eCDSS with individualized recommendations and any additional benefit of pharmacist outreach to follow-up compared with usual care. In this paper, we have reported the study design and initial implementation outcomes of this trial.

## Methods

### Overall Design

This was a 3-arm cluster randomized controlled trial with randomization at the provider level. There was 1 usual care control arm and 2 intervention arms. For patients of PCPs in the intervention arms, the research team ordered triple-marker testing (serum creatinine, serum cystatin-c, and urine ACR) at the beginning of the study period, to be completed when the patient visited the lab for regular clinical care. The eCDSS launched for PCPs and patients in the intervention arms during a regular PCP visit subsequent to completing the triple-marker testing. Patients in the eCDSS+ arm also received a pharmacist phone call to reinforce CKD-related education after a PCP visit in which the eCDSS was utilized. We planned for an 18-month trial beginning October 4, 2017, that included a 12-month intervention period and subsequent 6-month follow-up period. This trial was registered on ClinicalTrials.gov (NCT02925962). The University of California San Francisco Human Research Protection Program approved the protocol for this study.

### Setting

This study was conducted at a general internal medicine practice (with 2 locations) at the University of California San Francisco that cares for a diverse population of more than 24,000 adult patients. The PCPs in this practice include faculty attending physicians, resident physicians, and nurse practitioners (NPs). This practice is a certified primary care medical home (PCMH) that follows a team-based approach to care; each of the 10 teams includes 10 to 14 part-time PCPs, 2 medical assistants, a licensed vocational nurse, and administrative staff. Additional personnel of the PCMH include 5 registered nurses, 2 pharmacy technicians, and a clinical pharmacist available 1 day a week. The EHR used within this practice is Epic (EpicCare, Epic Systems).

### Pilot Activities

To optimize the chance of a successful intervention, we worked with a multidisciplinary team (general internist, informatics specialist, and nephrologists) to develop the eCDSS logic. The team focused on areas with the strongest evidence base for CKD management in primary care and developed the logic to apply guideline recommendations in real-time for individual patients. In the first step, patients were classified as either at high risk (eGFR_Cys_ <60 mL/min and/or ACR >30 mcg/mg) or low risk (eGFR_Cys_ >60 mL/min and ACR <30 mcg/mg) for experiencing CKD-related complications [5,17]. After risk stratification, the eCDSS logic focused on 5 domains of CKD care for high-risk patients: (1) cardiovascular risk reduction; (2) potassium management; (3) proteinuria management; (4) appropriateness for nephrology referral; and (5) patient education. For low-risk patients, the logic defaulted to a recommendation for repeat triple-marker testing in 6 months.

We elicited feedback from PCPs in focus group settings during development of the eCDSS and during implementation planning, which is a recommended practice to increase adoption and uptake of eCDSS [23]. Focus group discussions centered on eCDSS messaging and orders, as well as ways to facilitate integration into the usual clinical workflow.

### Eligibility and Selection of Participants

All providers practicing at the general internal medicine practice with a primary care panel were eligible for inclusion and randomization.

**Figure 1:**
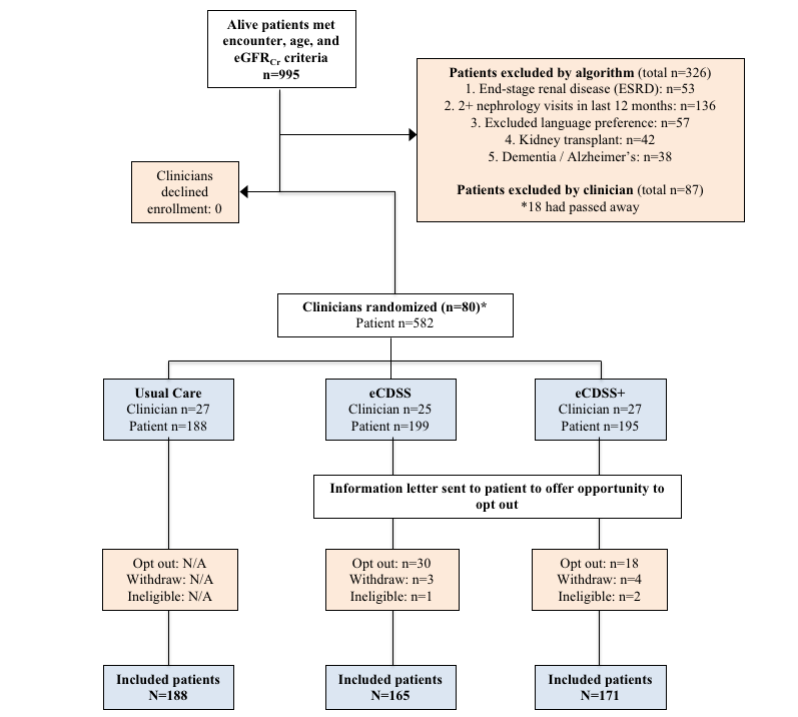
Flow diagram of participant selection. Note that 1 clinician who was randomized did not ultimately participate because their only eligible patient opted out. eGFR_Cr_: estimated glomerular filtration rate by serum creatinine; eCDSS: electronic clinical decision support system trial arm; eCDSS+: electronic clinical decision support system and pharmacist follow-up trial arm; N/A: not applicable.

Eligible patients were identified using a previously described and validated algorithm [40]. (Patients were considered eligible for inclusion if they were aged 18 to 80 years; had a preferred language of English, Spanish, or Chinese; had at least 2 outpatient eGFR_Cr_=30 to 59 milliliters/minute (mL/min) at least 90 days apart, one of which was within the 12 months before August 14, 2017 (when providers were first contacted for study recruitment); and had a primary care visit in the past 18 months. (see Figure 1 for the Consolidated Standards of Reporting Trial diagram) Participants were automatically excluded (based on EHR data) if they were deceased; diagnosed with end-stage renal disease; engaged with a nephrology clinic with 2 or more visits in the past 12 months; kidney transplant recipients; or diagnosed with dementia. PCPs were then sent a list of potential participants for inclusion and asked to exclude participants for additional criteria that were unreliably captured when extracted from the EHR: current pregnancy, life expectancy <6 months, limited communication ability owing to impaired cognition or severe mental illness, New York Heart Association Class III/IV heart failure, or known ejection fraction <25%. PCPs could also exclude any other participants they felt would be inappropriate for study staff to contact.

### Recruitment and Consent Process

We recruited PCPs with eligible patients by eGFR_Cr_ by email between August 14, 2017 and September 8, 2017. PCPs were given 2 weeks to either opt-out entirely or exclude individual patients. We then randomized participating PCPs as described below. Participation letters were mailed to eligible intervention arm patients between September 1, 2017 and September 27, 2017, and patients were given 2 weeks to either return an opt-out card or call the study coordinator to opt out. Patients opting out after the study start date of October 4, 2017, were considered withdrawn from the study.

The usual care group PCPs received no additional contact beyond recruitment from the study team, and the usual care patients were never contacted directly by the study team. Usual care PCPs did have access to the same labs and medications to use at their clinical discretion without any recommendations or direction from the study team.

### Randomization and Blinding

We utilized block randomization at the PCP level based on panel size. The study statistician randomized PCP participants to each of the 3 study arms using an automated procedure that accounted for cluster size, to assure balance both in terms of number of patients and number of providers per arm. Eligible patient participants were assigned to study arms based on their PCP’s assignment. The study statistician was blinded to the identification of the PCPs and patients and will be similarly blinded for the analyses.

### Intervention

Before the eCDSS rollout but after randomization, all participating PCPs randomized to an intervention arm were asked to watch an educational video that provided background on guideline-based CKD care, as well as information on what to expect when an enrolled patient participant came in for a visit. PCPs were given an incentive of US $10 and a chocolate bar upon watching the video. Overall, 69.8% (72.0% eCDSS and 67.9% eCDSS+) viewed the video.

Before the eCDSS rollout, between October 5, 2017 and October 12, 2017, one of the study investigators (LK) ordered triple-marker testing for each patient participant from the eCDSS arm (n=169) and each patient participant from the eCDSS+ arm (n=177). We excluded 48 patients who opted out. In total, 10 patients withdrew later.

**Figure 2:**
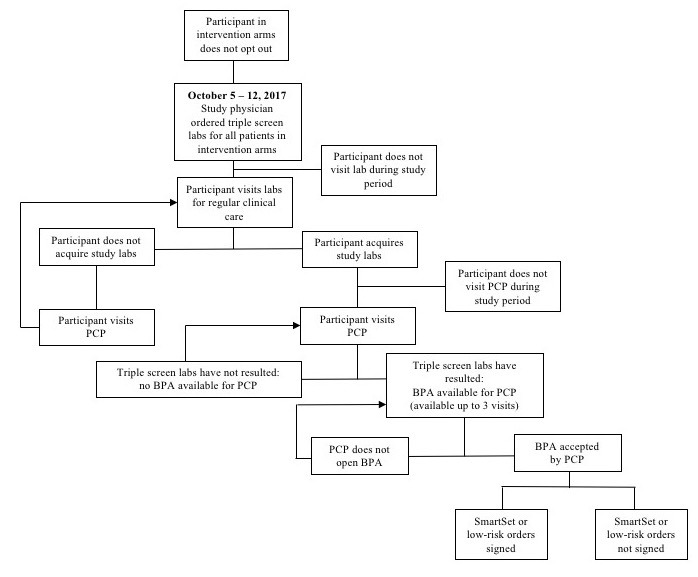
Flow diagram of study workflow for intervention arms. BPA: best practice advisory; PCP: primary care provider.

**Figure 3:**
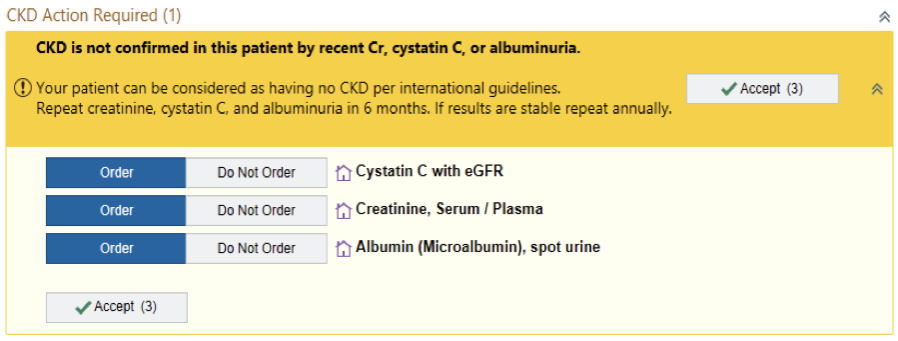
Electronic clinical decision support system trial arm low-risk best practice advisory. CKD: chronic kidney disease; Cr: serum creatinine; eGFR: estimated glomerular filtration rate.

**Figure 4:**
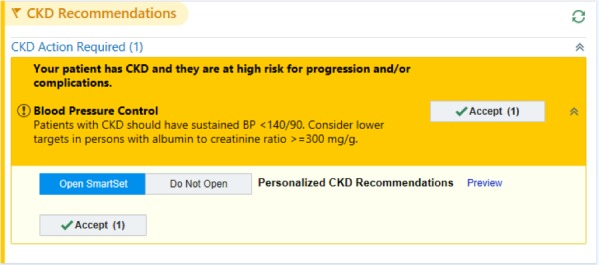
Electronic clinical decision support system trial arm high-risk best practice advisory. BP: blood pressure; CKD: chronic kidney disease; mg/g: milligrams per grams.

**Figure 5:**
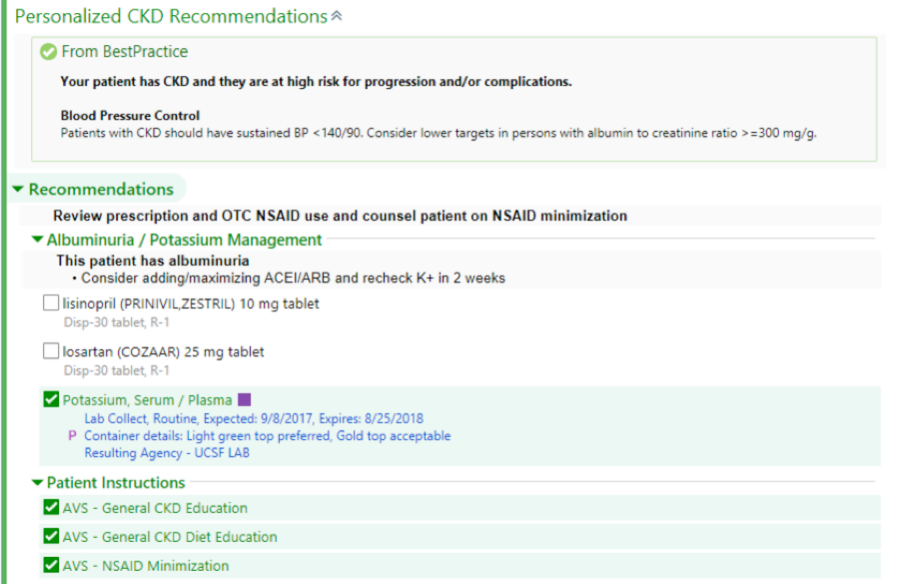
Electronic clinical decision support system trial arm example SmartSet. ACEi/ARB: angiotensin converting enzyme inhibitor / angiotensin II receptor blocker; AVS: after visit summary; CKD: chronic kidney disease; eCDSS: electronic clinical decision support system; CDSS+: electronic clinical decision support system and pharmacist follow-up trial arm; K+: potassium; mg/g: milligrams per grams; NSAID: nonsteroidal anti-inflammatory drug; OTC: over-the-counter;.

After the triple-marker testing was ordered, the next time the patient participant visited the lab for their regular clinical care, the triple-marker labs were also collected. We programmed the eCDSS to trigger the first time that the patient participant visited their PCP after all 3 lab results were available. (see Figure 2 for the study workflow) The eCDSS best practice advisory (BPA) appeared above the current medications in the electronic chart (Figures 3 and 4). PCPs could choose to open the BPA by clicking an “accept” button. We also allowed PCPs to view the recommendations by hovering over the BPA before accepting it. For patient participants categorized as low risk, when the PCP accepted the BPA, triple-marker labs with an expected completion date 6 months hence prepopulated were automatically pending for the PCP to sign. For patient participants categorized as high risk, when the PCP accepted the BPA, a SmartSet (EpicCare, Epic Systems) of tailored orders and recommendations appeared (Figure 5). If the PCP did not open the BPA and sign the orders in the SmartSet during the first PCP visit after the triple-marker labs were available, the BPA would trigger at up to 2 additional PCP visits during the study period. The eCDSS did not trigger if the patient participant saw a provider who was not their PCP.

In addition to a reminder of BP targets in CKD, the SmartSet for patient participants classified as high risk for progression and/or complications of CKD used real-time EHR lab and medication data to recommend any of the following that applied:

1. Cardiovascular risk reduction: recommendation for use of statin in all individuals aged ≥50 years not already on a statin, as recommended by Kidney Disease: Improving Global Outcomes (KDIGO) guidelines [17].
2. Potassium management: diet and diuretic recommendations.
3. Proteinuria management: initiation or titration of renin-angiotensin blockade with angiotensin converting enzyme inhibitors (ACEi) or angiotensin II receptor blockers (ARB).
4. Nephrology referral for highest risk participants: highest risk was defined as one of: confirmed eGFR_Cys_ <30 mL/min; potassium >5.5 mEq/L; ACR >300 mcg/mg, systolic blood pressure (SBP) >150 millimeters of mercury (mm Hg) despite 3 agents including a diuretic; >3% risk of five-year progression kidney failure based on Tangri equation [41].
5. Patient education: patient education materials to populate in the after-visit summary (AVS) focused on CKD general information, avoidance of nonsteroidal anti-inflammatory drugs (NSAIDs), and dietary recommendations.

The second intervention arm (eCDSS+) included a pharmacist follow-up visit by telephone. If the SmartSet was signed during the PCP visit, a pharmacist would call the participant within 2 weeks of the visit to reinforce CKD-related teaching and medication changes from the visit as well as complete a comprehensive medication review. Information on the telephone encounter was documented in the EHR and sent to the PCP; any urgent clinical issues identified during the call were highlighted for the PCP as needing their follow-up. Providers in the eCDSS+ group also were encouraged to make a warm handoff to the pharmacist by including anticipatory guidance about the pharmacist phone call within the after-visit summary and distributing a business card with the pharmacist’s photo and phone number for their patient participants at the time of using the SmartSet.

Given that the study physician ordered labs at the start of the intervention period, safety checks were put into place to ensure adequate review of the laboratory results. A nephrologist (LL) from the study team reviewed weekly laboratory results specifically to identify the following: eGFR_Cr_ decline >30% from baseline, ACR ≥1000 mcg/mg, adherence to nephrology referrals, and any discordance >30% between eGFR_Cr_ and eGFR_Cys._ In all of these situations, the nephrologist contacted the PCP to ensure appropriate follow-up. Specifically, in cases of discordance between the 2 types of eGFR levels, the nephrologist advised PCPs to dose medications based on eGFR­_Cys_ when the clinical scenario suggested that eGFR_cr_ may not be accurate.

### Strategies to Encourage Intervention and Behavior Change

Implementation studies have shown that interventions designed with a theoretical basis are more likely to be successful [42,43]. Therefore, we utilized several theory-based strategies to encourage provider uptake of the eCDSS and participant uptake of provider and/or pharmacist recommendations. We classify these strategies using the capability, opportunity, motivation, behavior framework (or COM-B), which asserts that capability, opportunity, and motivation are essential conditions that impact behavior [44,45].

To encourage provider uptake and use of CKD guidelines, we addressed *capability barriers* (such as knowledge about the guidelines or the eCDSS or forgetting to discuss CKD owing to limited bandwidth). During the recruitment phase, we educated eligible PCPs about the prevalence of CKD within their panel by sending a list of their potentially eligible participants along with information about the importance of recognizing and managing CKD early. Shortly before intervention implementation, we sent a training video about the CKD guidelines and the eCDSS to the participating intervention PCPs. The eCDSS itself also served as a prompt to discuss CKD during the visits.

To address PCP *opportunity barriers*, we designed the eCDSS to fit into the physician workflow and to contain all the necessary CKD-related orders and patient education in one easily accessible place during a clinical visit.

To address PCP *motivation barriers*, we provided a small incentive to the participating intervention PCPs who watched the training video. Finally, to enhance use of the BPA, study personnel sent an individual email reminder to the intervention PCP the day before each eligible patient participant visit.

Although the eCDSS itself was a provider-facing intervention, we used strategies to encourage patient participant behavior change. To address *capability barriers* (such as knowledge of CKD or forgetting the provider recommendations), the CDSS SmartSet had CKD-based patient educational handouts in the patient’s preferred language. The pharmacist phone call was also designed to reinforce the knowledge provided at the office visit and *motivate* the patient participant to adhere to medication and avoid NSAIDS.

### Data Collection

For patient participants, the EHR was used to collect baseline data about demographic characteristics (age, sex, race-ethnicity, preferred language, and insurance status); medical co-morbidities (cerebrovascular disease, congestive heart failure, coronary artery disease, diabetes mellitus, hyperlipidemia, and hypertension); current medication prescriptions (statin, ACEi/ARB, and diuretics); previous documentation of CKD on problem list and/or as a visit diagnosis, most recent blood pressure, eGFR­­_Cr,_ eGFR­­_Cys_ (if available), and urine ACR (if available) before enrollment.

Primary outcome data on BP will be extracted from the EHR. Baseline BP was defined as the most recent ambulatory BP measurement before final enrollment on October 4, 2017. All patient participant ambulatory BP measurements were collected during the intervention period and will continue to be collected for the 6 months after the end of the intervention.

Secondary outcomes will be collected primarily using automated EHR data extraction algorithms. The outcomes acquired through data extraction include all secondary outcomes except for provider and patient-centered outcomes, which are collected via surveys. Up to 25 participants in each of the 3 arms were surveyed to assess patient knowledge about CKD and NSAID avoidance. Patient participants in the eCDSS arm were surveyed 2 weeks after a visit in which the SmartSet was utilized; those in the eCDSS+ arm were surveyed 2 weeks after their pharmacist follow-up call, and those in the usual care arm were surveyed 2 weeks after a visit with their PCP during the study period. All participating intervention PCPs will be surveyed about their perception of the feasibility and utility of the eCDSS and pharmacist calls (for CDSS+ providers).

### Outcomes

#### Primary Outcome

A summary of pre-planned study outcomes is shown in Textbox 1. The primary clinical outcome of this study is BP change from baseline. This outcome will be assessed as a continuous variable (separately for both diastolic and systolic BP change from baseline) and dichotomous variable (sustained control, defined as BP <140/90 in 2 or more consecutive visits during the trial). As this study was initiated before the 2017 BP guidelines, <140/90 was used to define control; this value also aligns with current KDIGO recommendations [17,46]. Rates of control at the end of the intervention period and 6 months later will be assessed based on the most recent BP measurement carried forward.

**Outcome measures. ACEi/ARB: angiotensin converting enzyme inhibitor / angiotensin II receptor blocker; BPA: best practice advisory; CKD: chronic kidney disease; PCP: primary care provider; NSAID: nonsteroidal anti-inflammatory drug.**

**Primary outcome:** blood pressure

- Systolic blood pressure change
- Diastolic blood pressure change
- Controlled blood pressure (defined as <140/90)

**Secondary outcomes**

- Process
  - PCP awareness of CKD diagnosis (CKD inclusion on problem list or visit diagnosis)
  - ACEi/ARB utilization for albuminuria
  - Statin therapy for eligible high-risk CKD patients
    - Initiation of statin therapy
    - Total use of statin therapy
- Patient-centered
  - Knowledge about CKD
  - Awareness of NSAID avoidance in CKD

**Implementation outcomes**

- Reach
  - Recruitment rate of providers and patients
  - Proportion that complete triple marker screen
  - Proportion with PCP visit after complete triple marker screening
  - Proportion of participants where BPA is available for PCP
  - Proportion of participants identified as high- vs low-risk CKD
- Adoption
  - Proportion where PCP signed Smart Set when BPA was available
  - Proportion of CDSS+ participants that received pharmacist phone call after PCP signed Smart Set
- Implementation
  - Proportion of orders signed or patient education provided by PCP after opening Smart Set
  - Proportion referred to nephrology and followed up with nephrology after signing Smart Set
  - Proportion of low-risk CKD patients that receive repeat triple screen
- Maintenance
  - PCP satisfaction with eCDSS
  - PCP intent to continue using eCDSS

#### Secondary Outcomes

Our main secondary outcome is PCP awareness of the patient participant’s CKD diagnosis, as measured by inclusion of CKD-related International Statistical Classification of Diseases and Related Health Problems-10th Revision codes on the problem list or as a visit diagnosis. Additional secondary outcomes include process of care, patient-centered, and implementation outcomes.

There are 2 main process of care outcomes: (1) the proportion of patient participants with albuminuria prescribed ACEi/ARB and (2) appropriate use of statins. Appropriate statin use is defined both as the proportion of all patient participants aged ≥50 years prescribed statin therapy and the proportion of participants initiated on statin therapy after study start date.

Patient-centered outcomes include patient knowledge about CKD and NSAID avoidance as measured by patient surveys.

In addition to the effectiveness outcomes described above, the implementation outcomes are described in detail in Textbox 1 using the reach, effectiveness, adoption, implementation, and maintenance (or RE-AIM) framework to highlight measures crucial to successful implementation [47]. In this paper, we report initial implementation results, including all of the Reach outcomes and the first Adoption outcome (PCP SmartSet use).

### Analyses

Baseline demographic and clinical characteristics will be summarized and study balance by study arm will be assessed using descriptive statistics as well as methods that account for the lack of independence among patients in the same cluster [48]. Balance will be assessed with logit link generalized estimating equation (GEE) for categorical variables and linear GEE models for continuous variables. Clustering will be accounted for using an exchangeable correlation matrix with robust standard errors. We anticipate controlling for characteristics that are associated with outcomes but differ at baseline between arms and using multivariable regression analyses with GEE to account for cluster randomization in the reporting of the final outcomes of our study. Sensitivity analyses will include *as treated* analyses with inverse probability weighting to determine impact of participants changing providers/study arms as well as to determine impact of any differences in the number of assessments or clinic visits by study arm. Primary analyses will follow intention-to-treat principles.

### Sample Size and Power Calculation

Based on preliminary data from our institution’s EHR, it was determined that there would be a maximum of 1400 participants in the practice who could meet inclusion criteria. The study was powered for the clinical outcome of BP change, with calculations performed using the clustersampsi command for Stata version 11.2. We assumed a 2-tailed alpha level of 0.05, an intraclass correlation coefficient of 0.025 based on studies in primary care settings [49,50], and a standard deviation of 5 mmHg. Assuming 23 clinicians per arm with at least 15 patients per clinician (ie, 345 patients per arm), we estimated that we would have 80% power to detect a difference as small as 1.27 mmHg between arms.

## Results

### Implementation Outcomes: Reach

Implementation outcomes at the end of the 12-month intervention period on October 4, 2018, are summarized below:

#### Recruitment Rate

At the provider level, all 81 eligible PCPs (47 faculty attending physicians, 31 resident physicians, and 3 NPs) agreed to participate (100%). In total, 79 of the 81 providers (98%) were included because 2 providers had no remaining eligible participants after exclusion and opt-outs. At the patient level, 995 patients who met the initial eligibility criteria were identified. A total of 316 / 995 (31.8%) potential patient participants were excluded using automated algorithms, and clinicians excluded another 90 patients (Figure 1). A total of 582 patient participants were distributed to the 3 arms based on provider randomization. After an additional 3 patients were excluded (owing to patient deaths not recorded in EHR before randomization) and 55 / 582 patients (9.5%) opted out or withdrew, 524 patient participants (90.0% of eligible 582 participants) were included in the study: 188 usual care, 165 eCDSS, and 171 eCDSS+.

Baseline patient participant characteristics are shown in Table 1. The 55 patients that opted out/withdrew were similar to the patient participants across multiple traits—including age, gender, race/ethnicity, language preference, and co-morbidities—except they had lower renal function on average: eGFR_Cr_ (52.7 [SD 11.7] versus 56.0 [SD 11.8]; *P*=.046).

**Table 1:** Baseline characteristics of all included patients (N=524).

Patient characteristic		Values
Age (years), mean (SD)		70.3 (8.9)
Female, n (%)		236 (45)
**Race/ethnicity, n (%)**		
	White	277 (53)
	Asian	118 (23)
	Black/African American	70 (13)
	Hispanic	38 (7)
	Other	20 (4)
**Preferred language, n (%)**		
	English	469 (90)
	Chinese	39 (7)
	Spanish	16 (3)
**Insurance, n (%)**		
	Private insurance	146 (28)
	Medicare	215 (41)
	Medicaid	163 (31)
**Medical co-morbidities, n (%)**		
	Cerebrovascular disease	36 (7)
	Congestive heart failure	40 (8)
	Coronary artery disease	95 (18)
	Diabetes mellitus	199 (38)
	Hyperlipidemia	302 (58)
	Hypertension	377 (72)
**Medication use, n (%)**		
	Angiotensin converting enzyme inhibitor/angiotensin II receptor blocker	319 (61)
	Diuretic	199 (38)
	Statin	353 (67)
**CKD-related outcomes, n (%)**		
	Chronic kidney disease (CKD) on problem list	61 (12)
	CKD on problem list or primary care visit diagnosis by ICD-10^c^ codes	247 (47)
Systolic BP^a^ (mm Hg), median (Q1-Q3)		128 (117-140)
Diastolic BP (mm Hg), median (Q1-Q3)		68 (62.5-75)
BP controlled^b^ (<140/90), n (%)		373 (71)
Estimated glomerular filtration rate based by serum creatinine (mL/min), mean (SD)		56.0 (11.8)

^a^BP: blood pressure.

^b^BP control reported based on only the most recent measurement before the start of the study.

^c^ICD-10: International Classification of Diseases, Tenth Revision, Clinical Modification.

#### Completion of Triple Marker

Of the 336 patient participants in the intervention arms, 178 (53.0%) completed the triple-marker screening.

#### PCP Visits

Of the 178 patient participants who completed a triple-marker screen, 138 (77.5%) had a PCP visit during the 12-month intervention period. This was 41.1% (138/336) of all intervention participants.

#### PCP BPA Use

Of the 138 participants with a PCP visit, the BPA was opened for 102 participants (73.9%). This was 30.3% (102/336) of all intervention participants.

### Implementation Outcomes: Adoption

#### SmartSet Use

Of the 102 participants in which the BPA was opened, orders were signed or patient education was given from the SmartSet for 83 participants (81.4%).

## Discussion

### Principal Findings

In this report, we describe the rationale, design, and initial implementation outcomes of a 3-arm pragmatic trial that assessed the feasibility and preliminary effectiveness of an eCDSS to improve CKD management in primary care compared with usual care. This study builds on previous pragmatic trials focused on EHR-based interventions to improve CKD management [28,29,51,52] and supports the feasibility of using EHRs to identify study participants, intervene in early CKD management, and measure study outcomes. Given its design as a pragmatic trial (per Pragmatic Explanatory Continuum Indicator Summary, or PRECIS, criteria [53]), we found high rates of participation by providers and low opt-out rates by patients. We also found high rates of eCDSS use by providers.

This study has many innovative components. We utilized a 3-armed trial design, which included a usual care group as well as a group with pharmacist-patient engagement after the provider-facing electronic intervention. In addition, our clinical eCDSS was mostly automated and individualized to patients based on laboratory results, rather than a generic CDSS for all participants. We also implemented the recommended triple-marker risk stratification approach to better characterize participants’ risk for CKD progression. Our participants included non-English speakers, who have been excluded from many other trials. This trial also included multiple types of outcomes allowing for a deeper understanding of the intervention’s impact on care processes. Reviews of eCDSS have found that few studies assess unintended consequences or adverse effects of a CDSS, which can be widespread as CDSS interventions often automate multiple actions [54,55]. This trial integrated safety measures and their tracking by a nephrologist allowing not only for measurement of safety but also the ability to ensure that any new concerning clinical findings found owing to the trial would be addressed. As previously recommended, it is important to first pilot interventions before widespread implementation to ensure there are no adverse effects in a small population where safety can be more easily assessed [56].

One of the greatest challenges of this trial was its dependence on data analysts experienced at working with the EHR to program and implement the intervention as well as extract outcomes data. The study team devoted substantial time to troubleshoot unanticipated problems with the eCDSS programming and capture of outcomes. Every update or change to the EHR processes had the potential to disrupt the trial implementation. The team regularly validated the eCDSS and data capture through manual chart reviews. The inaccuracies of EHR data extraction are well-documented [57]. Despite the presence of an explicit trial infrastructure in our EHR, implementation of the intervention and data collection was far from seamless. Studies that rely on the EHR to deliver interventions and collect outcomes must ensure adequate time and resources for a multidisciplinary team to validate the intervention and review data inaccuracies in an ongoing manner for all EHR-based trials.

### Comparison With Previous Work

In this trial, we found much higher reach and adoption of eCDSS by PCPs than previous studies where adoption rates were frequently <50% [55,58]. We believe there are multiple explanations. Few electronic alerts are currently used in this practice, so providers have not yet experienced fatigue associated with the growing presence of alerts [59]. Moreover, the eCDSS was designed by a multidisciplinary team, facilitating buy-in from all stakeholders and ensuring all perspectives were considered in determining how the eCDSS would impact workflow. Finally, study staff (including a lead investigator who works within the primary care practice) served as on-the-ground champions and made significant efforts to educate, remind, and incentivize PCPs.

We experienced many of the same challenges as other pragmatic trials during implementation of this EHR-dependent intervention. There was a period of time between provider recruitment, randomization, participants being contacted to opt out, and the study start date; as a result, 18 patients passed away before randomization and 2 additional patients passed away before the start of the study. Importantly, nearly half of the participants in the intervention arms never completed the triple-marker screen and therefore never had the opportunity to receive the eCDSS interventions. Another one-fifth of participants who completed the triple-marker screen did not have a PCP visit after the triple-marker screening and also did not receive the eCDSS. These barriers, common in many eCDSS trials, prevented the intervention group from receiving the desired intervention.

### Limitations

As a pilot study, this study was limited by its inclusion of patients and providers from a single institution. Therefore, both the intervention and strategies to encourage intervention uptake were adapted to meet the needs of this single institution that uses Epic Systems’ EHR. However, we have found that the patient population in this clinic is similar to others [60], and Epic is a widely used EHR in multiple health systems. As a pragmatic trial, our sample size was smaller than initially anticipated and will thus ultimately impact our power to detect changes in our primary outcome. The short follow-up period for this pilot study will also limit our ability to assess some clinical outcomes. However, we were still able to use this pilot study to determine the feasibility of this intervention.

### Conclusions

As the prevalence of CKD grows, primary care teams will increasingly be responsible for management of CKD. Clinical decision support tools may be a low-cost effective solution to enhance guideline-concordant care for this underdiagnosed disease. We describe the design and implementation of a pragmatic clustered randomized controlled trial to evaluate the feasibility and effectiveness an EHR-embedded electronic clinical decision support tool to improve management of CKD in primary care. Results from this study can guide design of future pragmatic eCDSS trials to improve CKD care.

## Abbreviations

ACEi: angiotensin converting enzyme inhibitor
ACR: urine albumin-creatinine ratio
ARB: angiotensin II receptor blocker
BP: blood pressure
BPA: best practice advisory
CKD: chronic kidney disease
eCDSS: electronic clinical decision support system trial arm
eCDSS+: electronic clinical decision support system and pharmacist follow-up trial arm
eGFR: estimated glomerular filtration rate
eGFR _Cr_: estimated glomerular filtration rate by serum creatinine
eGFR _Cys_: estimated glomerular filtration rate by serum cystatin-c
EHR: electronic health record
GEE: generalized estimating equation
ICD-10: International Classification of Diseases, Tenth Revision, Clinical Modification
KDIGO: Kidney Disease: Improving Global Outcomes
NP: nurse practitioner
NSAID: nonsteroidal anti-inflammatory drug
PCMH: primary care medical home
PCP: primary care provider

## Appendix

Multimedia Appendix 1 CONSORT‐EHEALTH checklist (V 1.6.1).
