# Supplementary material for: A Pragmatic Cluster Randomized Trial of an Electronic Clinical Decision Support System to Improve Chronic Kidney Disease Management in Primary Care: Design, Rationale, and Implementation Experience
Source: JMIR Res Protoc. 2019 Jun 7;8(6):e14022. doi: 10.2196/14022 (PMC6594214; doi:10.2196/14022)
Supplement: Multimedia Appendix 1 [file resprot_v8i6e14022_app1.pdf]

CONSORT-EHEALTH (V 1.6.1) - Submission/Publication Form

The CONSORT-EHEALTH checklist is intended for authors of randomized trials evaluating web-based and Internet-based applications/interventions, including mobile interventions, electronic games (incl multiplayer games), social media, certain telehealth applications, and other interactive and/or networked electronic applications. Some of the items (e.g. all subitems under item 5 - description of the intervention) may also be applicable for other study designs.

The goal of the CONSORT EHEALTH checklist and guideline is to be
a) a guide for reporting for authors of RCTs,
b) to form a basis for appraisal of an ehealth trial (in terms of validity)

CONSORT-EHEALTH items/subitems are MANDATORY reporting items for studies published in the Journal of Medical Internet Research and other journals / scientific societies endorsing the checklist.

Items numbered 1., 2., 3., 4a., 4b etc are original CONSORT or CONSORT-NPT (non-pharmacologic treatment) items.
Items with Roman numerals (i., ii, iii, iv etc.) are CONSORT-EHEALTH extensions/clarifications.

As the CONSORT-EHEALTH checklist is still considered in a formative stage, we would ask that you also RATE ON A SCALE OF 1-5 how important/useful you feel each item is FOR THE PURPOSE OF THE CHECKLIST and reporting guideline (optional).

Mandatory reporting items are marked with a red *.
In the textboxes, either copy & paste the relevant sections from your manuscript into this form - please include any quotes from your manuscript in QUOTATION MARKS,
or answer directly by providing additional information not in the manuscript, or elaborating on why the item was not relevant for this study.

YOUR ANSWERS WILL BE PUBLISHED AS A SUPPLEMENTARY FILE TO YOUR PUBLICATION IN JMIR AND ARE CONSIDERED PART OF YOUR PUBLICATION (IF ACCEPTED).
Please fill in these questions diligently. Information will not be copyedited, so please use proper spelling and grammar, use correct capitalization, and avoid abbreviations.

DO NOT FORGET TO SAVE AS PDF _AND_ CLICK THE SUBMIT BUTTON SO YOUR ANSWERS ARE IN OUR DATABASE !!!

Citation Suggestion (if you append the pdf as Appendix we suggest to cite this paper in the caption):
Eysenbach G, CONSORT-EHEALTH Group
CONSORT-EHEALTH: Improving and Standardizing Evaluation Reports of Web-based and Mobile Health Interventions
J Med Internet Res 2011;13(4):e126
URL: http://www.jmir.org/2011/4/e126/
doi: 10.2196/jmir.1923
PMID: 22209829

* Required

Your name *

First Last

Elaine Khoong

Primary Affiliation (short), City, Country *

University of Toronto, Toronto, Canada

UCSF DGIM at ZSFG, San Francisco, CA

Your e-mail address *

abc@gmail.com

elaine.khoong@ucsf.edu

Title of your manuscript *

Provide the (draft) title of your manuscript.

A Pragmatic Cluster Randomized Trial of an Electronic Clinical Decision Support System to Improve Chronic Kidney Disease Management in Primary Care: Design, Rationale, and Implementation Experience

Name of your App/Software/Intervention *

If there is a short and a long/alternate name, write the short name first and add the long name in brackets.

eCDSS (electronic clinical decision suppor

Evaluated Version (if any)

e.g. "V1", "Release 2017-03-01", "Version 2.0.27913"

Your answer

Language(s) *

What language is the intervention/app in? If multiple languages are available, separate by comma (e.g. "English, French")

English

URL of your Intervention Website or App

e.g. a direct link to the mobile app on app in appstore (itunes, Google Play), or URL of the website. If the intervention is a DVD or hardware, you can also link to an Amazon page.

Your answer

URL of an image/screenshot (optional)

Your answer

Accessibility *

Can an enduser access the intervention presently?

- ○ access is free and open
- ◉ access only for special usergroups, not open
- ○ access is open to everyone, but requires payment/subscription/in-app purchases
- ○ app/intervention no longer accessible
- ○ Other:

Primary Medical Indication/Disease/Condition *

e.g. "Stress", "Diabetes", or define the target group in brackets after the condition, e.g. "Autism (Parents of children with)", "Alzheimers (Informal Caregivers of)"

Chronic Kidney Disease

Primary Outcomes measured in trial *

comma-separated list of primary outcomes reported in the trial

"The primary clinical outcome of this stud

Secondary/other outcomes

Are there any other outcomes the intervention is expected to affect?

"Our main secondary outcome is PCP awareness of the patient participant’s CKD diagnosis...." "Additional secondary outcomes include process of care, patient-centered, and implementation outcomes." Please additionally see Figure 6 of all planned outcomes.

Recommended "Dose" *

What do the instructions for users say on how often the app should be used?

- ○ Approximately Daily
- ○ Approximately Weekly
- ○ Approximately Monthly
- ○ Approximately Yearly
- ○ "as needed"
- ◉ Other: To be used by providers when they see patients within their clinic who m

Approx. Percentage of Users (starters) still using the app as recommended after 3 months *

- (selected) unknown / not evaluated
- 0-10%
- 11-20%
- 21-30%
- 31-40%
- 41-50%
- 51-60%
- 61-70%
- 71%-80%
- 81-90%
- 91-100%
- Other:

Overall, was the app/intervention effective? *

- ( ) yes: all primary outcomes were significantly better in intervention group vs control
- ( ) partly: SOME primary outcomes were significantly better in intervention group vs control
- ( ) no statistically significant difference between control and intervention
- ( ) potentially harmful: control was significantly better than intervention in one or more outcomes
- ( ) inconclusive: more research is needed
- (•) Other: This is a protocol study. We have not collected primary outcomes yet.

Article Preparation Status/Stage *

At which stage in your article preparation are you currently (at the time you fill in this form)

- (•) not submitted yet - in early draft status
- ( ) not submitted yet - in late draft status, just before submission
- ( ) submitted to a journal but not reviewed yet
- ( ) submitted to a journal and after receiving initial reviewer comments
- ( ) submitted to a journal and accepted, but not published yet
- ( ) published
- ( ) Other:

Journal *

If you already know where you will submit this paper (or if it is already submitted), please provide the journal name (if it is not JMIR, provide the journal name under "other")

- (●) not submitted yet / unclear where I will submit this
- ( ) Journal of Medical Internet Research (JMIR)
- ( ) JMIR mHealth and UHealth
- ( ) JMIR Serious Games
- ( ) JMIR Mental Health
- ( ) JMIR Public Health
- ( ) JMIR Formative Research
- ( ) Other JMIR sister journal
- ( ) Other:

Is this a full powered effectiveness trial or a pilot/feasibility trial? *

- (●) Pilot/feasibility
- ( ) Fully powered

Manuscript tracking number *

If this is a JMIR submission, please provide the manuscript tracking number under "other" (The ms tracking number can be found in the submission acknowledgement email, or when you login as author in JMIR. If the paper is already published in JMIR, then the ms tracking number is the four-digit number at the end of the DOI, to be found at the bottom of each published article in JMIR)

- ( ) no ms number (yet) / not (yet) submitted to / published in JMIR
- (●) Other: The protocol for this study is accepted for publication in JMIR research

TITLE AND ABSTRACT

1a) TITLE: Identification as a randomized trial in the title

1a) Does your paper address CONSORT item 1a? *

I.e does the title contain the phrase "Randomized Controlled Trial"? (if not, explain the reason under "other")

- (selected) yes
- Other:

1a-i) Identify the mode of delivery in the title

Identify the mode of delivery. Preferably use “web-based” and/or “mobile” and/or “electronic game” in the title. Avoid ambiguous terms like “online”, “virtual”, “interactive”. Use “Internet-based” only if Intervention includes non-web-based Internet components (e.g. email), use “computer-based” or “electronic” only if offline products are used. Use “virtual” only in the context of “virtual reality” (3-D worlds). Use “online” only in the context of “online support groups”. Complement or substitute product names with broader terms for the class of products (such as “mobile” or “smart phone” instead of “iphone”), especially if the application runs on different platforms.

	1	2	3	4	5	
subitem not at all important	○	○	○	●	○	essential

Does your paper address subitem 1a-i? *

Copy and paste relevant sections from manuscript title (include quotes in quotation marks "like this" to indicate direct quotes from your manuscript), or elaborate on this item by providing additional information not in the ms, or briefly explain why the item is not applicable/relevant for your study

"A Pragmatic Cluster Randomized Trial of an Electronic Clinical Decision Support System..."

1a-ii) Non-web-based components or important co-interventions in title

Mention non-web-based components or important co-interventions in title, if any (e.g., “with telephone support”).

	1	2	3	4	5	
subitem not at all important	○	○	○	○	○	essential

Does your paper address subitem 1a-ii?

Copy and paste relevant sections from manuscript title (include quotes in quotation marks "like this" to indicate direct quotes from your manuscript), or elaborate on this item by providing additional information not in the ms, or briefly explain why the item is not applicable/relevant for your study

Not in title, but described within the protocol (the pharmacist outreach in the eCDSS+ intervention arm)

1a-iii) Primary condition or target group in the title

Mention primary condition or target group in the title, if any (e.g., “for children with Type I Diabetes”) Example: A Web-based and Mobile Intervention with Telephone Support for Children with Type I Diabetes: Randomized Controlled Trial

	1	2	3	4	5	
subitem not at all important	○	○	○	○	◉	essential

Does your paper address subitem 1a-iii? *

Copy and paste relevant sections from manuscript title (include quotes in quotation marks "like this" to indicate direct quotes from your manuscript), or elaborate on this item by providing additional information not in the ms, or briefly explain why the item is not applicable/relevant for your study

"A Pragmatic Cluster Randomized Trial of an Electronic Clinical Decision Support System to Improve Chronic Kidney Disease Management in Primary Care"

1b) ABSTRACT: Structured summary of trial design, methods, results, and conclusions

NPT extension: Description of experimental treatment, comparator, care providers, centers, and blinding status.

1b-i) Key features/functionalities/components of the intervention and comparator in the METHODS section of the ABSTRACT

Mention key features/functionalities/components of the intervention and comparator in the abstract. If possible, also mention theories and principles used for designing the site. Keep in mind the needs of systematic reviewers and indexers by including important synonyms. (Note: Only report in the abstract what the main paper is reporting. If this information is missing from the main body of text, consider adding it)

	1	2	3	4	5	
subitem not at all important	○	○	○	◉	○	essential

Does your paper address subitem 1b-i? *

Copy and paste relevant sections from the manuscript abstract (include quotes in quotation marks "like this" to indicate direct quotes from your manuscript), or elaborate on this item by providing additional information not in the ms, or briefly explain why the item is not applicable/relevant for your study

"This was a 3-arm pragmatic cluster-randomized trial at an academic general internal medicine practice." "The eCDSS launched for PCPs and patients in the intervention arms during a regular PCP visit subsequent to completing the triple-marker testing. The eCDSS delivered individualized guidance on cardiovascular risk-reduction, potassium and proteinuria management, and patient education. Patients in the eCDSS+ arm also received a pharmacist phone call to reinforce CKD-related education."

1b-ii) Level of human involvement in the METHODS section of the ABSTRACT

Clarify the level of human involvement in the abstract, e.g., use phrases like “fully automated” vs. “therapist/nurse/care provider/physician-assisted” (mention number and expertise of providers involved, if any). (Note: Only report in the abstract what the main paper is reporting. If this information is missing from the main body of text, consider adding it)

	1	2	3	4	5	
subitem not at all important	○	○	○	◉	○	essential

Does your paper address subitem 1b-ii?

Copy and paste relevant sections from the manuscript abstract (include quotes in quotation marks "like this" to indicate direct quotes from your manuscript), or elaborate on this item by providing additional information not in the ms, or briefly explain why the item is not applicable/relevant for your study

"This was a 3-arm pragmatic cluster-randomized trial at an academic general internal medicine practice." "The eCDSS launched for PCPs and patients in the intervention arms during a regular PCP visit subsequent to completing the triple-marker testing. The eCDSS delivered individualized guidance on cardiovascular risk-reduction, potassium and proteinuria management, and patient education. Patients in the eCDSS+ arm also received a pharmacist phone call to reinforce CKD-related education."

1b-iii) Open vs. closed, web-based (self-assessment) vs. face-to-face assessments in the METHODS section of the ABSTRACT

Mention how participants were recruited (online vs. offline), e.g., from an open access website or from a clinic or a closed online user group (closed usergroup trial), and clarify if this was a purely web-based trial, or there were face-to-face components (as part of the intervention or for assessment). Clearly say if outcomes were self-assessed through questionnaires (as common in web-based trials). Note: In traditional offline trials, an open trial (open-label trial) is a type of clinical trial in which both the researchers and participants know which treatment is being administered. To avoid confusion, use “blinded” or “unblinded” to indicated the level of blinding instead of “open”, as “open” in web-based trials usually refers to “open access” (i.e. participants can self-enrol). (Note: Only report in the abstract what the main paper is reporting. If this information is missing from the main body of text, consider adding it)

	1	2	3	4	5	
subitem not at all important	○	○	○	◉	○	essential

Does your paper address subitem 1b-iii?

Copy and paste relevant sections from the manuscript abstract (include quotes in quotation marks "like this" to indicate direct quotes from your manuscript), or elaborate on this item by providing additional information not in the ms, or briefly explain why the item is not applicable/relevant for your study

"This was a 3-arm pragmatic cluster-randomized trial at an academic general internal medicine practice. Eligible patients had 2 previous estimated-glomerular-filtration-rates by serum creatinine (eGFRCr) <60 mL/min/1.73m2 at least 90 days apart. Randomization occurred at the PCP level. For patients of PCPs in either of the 2 intervention arms, the research team ordered triple-marker testing (serum creatinine, serum cystatin-c, and urine albumin-creatinine-ratio) at the beginning of the study period, to be completed when acquiring labs for regular clinical care. The eCDSS launched for PCPs and patients in the intervention arms during a regular PCP visit subsequent to completing the triple-marker testing. The eCDSS delivered individualized guidance on cardiovascular risk-reduction, potassium and proteinuria management, and patient education. Patients in the eCDSS+ arm also received a pharmacist phone call to reinforce CKD-related education."

1b-iv) RESULTS section in abstract must contain use data

Report number of participants enrolled/assessed in each group, the use/uptake of the intervention (e.g., attrition/adherence metrics, use over time, number of logins etc.), in addition to primary/secondary outcomes. (Note: Only report in the abstract what the main paper is reporting. If this information is missing from the main body of text, consider adding it)

	1	2	3	4	5	
subitem not at all important	○	○	○	○	○	essential

Does your paper address subitem 1b-iv?

Copy and paste relevant sections from the manuscript abstract (include quotes in quotation marks "like this" to indicate direct quotes from your manuscript), or elaborate on this item by providing additional information not in the ms, or briefly explain why the item is not applicable/relevant for your study

"All 81 PCPs contacted agreed to participate and were randomized. Of 995 patients initially eligible by eGFRCr, 413 were excluded per protocol and 58 opted out or withdrew, resulting in 524 patient participants (188 usual care; 165 eCDSS; and 171 eCDSS+). During the 12-month intervention period, 53.0% (178/336) of intervention patient participants completed triple-marker labs. Among these, 138/178 (77.5%) had a PCP appointment after the triple-marker labs resulted; the eCDSS was opened for 73.9% (102/138), with orders or education signed for 81.4% (83/102)."

1b-v) CONCLUSIONS/DISCUSSION in abstract for negative trials

Conclusions/Discussions in abstract for negative trials: Discuss the primary outcome - if the trial is negative (primary outcome not changed), and the intervention was not used, discuss whether negative results are attributable to lack of uptake and discuss reasons. (Note: Only report in the abstract what the main paper is reporting. If this information is missing from the main body of text, consider adding it)

	1	2	3	4	5	
subitem not at all important	○	○	○	○	○	essential

Does your paper address subitem 1b-v?

Copy and paste relevant sections from the manuscript abstract (include quotes in quotation marks "like this" to indicate direct quotes from your manuscript), or elaborate on this item by providing additional information not in the ms, or briefly explain why the item is not applicable/relevant for your study

No; this is a protocol paper. Primary outcome of trial not reported.

INTRODUCTION

2a) In INTRODUCTION: Scientific background and explanation of rationale

2a-i) Problem and the type of system/solution

Describe the problem and the type of system/solution that is object of the study: intended as stand-alone intervention vs. incorporated in broader health care program? Intended for a particular patient population? Goals of the intervention, e.g., being more cost-effective to other interventions, replace or complement other solutions? (Note: Details about the intervention are provided in “Methods” under 5)

	1	2	3	4	5	
subitem not at all important	○	○	○	○	○	essential

Does your paper address subitem 2a-i? *

Copy and paste relevant sections from the manuscript (include quotes in quotation marks "like this" to indicate direct quotes from your manuscript), or elaborate on this item by providing additional information not in the ms, or briefly explain why the item is not applicable/relevant for your study

"Given the promise of both eCDSS and team-based care to improve guideline-concordant CKD care in primary care practice, we designed a pragmatic cluster randomized 3-arm trial (usual care; eCDSS; and eCDSS plus pharmacist follow-up). This trial aimed to assess the feasibility and impact of a CKD eCDSS with individualized recommendations and any additional benefit of pharmacist outreach to follow-up compared with usual care."

2a-ii) Scientific background, rationale: What is known about the (type of) system

Scientific background, rationale: What is known about the (type of) system that is the object of the study (be sure to discuss the use of similar systems for other conditions/diagnoses, if appropiate), motivation for the study, i.e. what are the reasons for and what is the context for this specific study, from which stakeholder viewpoint is the study performed, potential impact of findings [2]. Briefly justify the choice of the comparator.

	1	2	3	4	5	
subitem not at all important	○	○	○	○	○	essential

Does your paper address subitem 2a-ii? *

Copy and paste relevant sections from the manuscript (include quotes in quotation marks "like this" to indicate direct quotes from your manuscript), or elaborate on this item by providing additional information not in the ms, or briefly explain why the item is not applicable/relevant for your study

"CKD identification and management is an ideal area for targeted electronic efforts [22,23]. Previous studies have shown the feasibility of electronic clinical decision support systems (eCDSS) to improve guideline-concordant care for patients with CKD [24-29]. However, most studies have not been randomized
nor have they provided individualized recommendations for patients based on laboratory results. Instead, they have utilized standard reminders or checklists. In addition, concerns about the eCDSS being burdensome or disruptive to workflow have hindered the development and testing of more complex,
individualized eCDSS"

2b) In INTRODUCTION: Specific objectives or hypotheses

Does your paper address CONSORT subitem 2b? *

Copy and paste relevant sections from the manuscript (include quotes in quotation marks "like this" to indicate direct quotes from your manuscript), or elaborate on this item by providing additional information not in the ms, or briefly explain why the item is not applicable/relevant for your study

"This trial aimed to assess the feasibility and impact of a CKD eCDSS with individualized recommendations and any additional benefit of pharmacist outreach to follow-up compared with usual care."

METHODS

3a) Description of trial design (such as parallel, factorial) including allocation ratio

Does your paper address CONSORT subitem 3a? *

Copy and paste relevant sections from the manuscript (include quotes in quotation marks "like this" to indicate direct quotes from your manuscript), or elaborate on this item by providing additional information not in the ms, or briefly explain why the item is not applicable/relevant for your study

"This was a 3-arm cluster randomized controlled trial with randomization at the provider level. There was 1 usual care control arm and 2 intervention arms. "

3b) Important changes to methods after trial commencement (such as eligibility criteria), with reasons

Does your paper address CONSORT subitem 3b? *

Copy and paste relevant sections from the manuscript (include quotes in quotation marks "like this" to indicate direct quotes from your manuscript), or elaborate on this item by providing additional information not in the ms, or briefly explain why the item is not applicable/relevant for your study

No major trial changes

3b-i) Bug fixes, Downtimes, Content Changes

Bug fixes, Downtimes, Content Changes: ehealth systems are often dynamic systems. A description of changes to methods therefore also includes important changes made on the intervention or comparator during the trial (e.g., major bug fixes or changes in the functionality or content) (5-iii) and other “unexpected events” that may have influenced study design such as staff changes, system failures/downtimes, etc. [2].

	1	2	3	4	5	
subitem not at all important	○	○	○	○	○	essential

Does your paper address subitem 3b-i?

Copy and paste relevant sections from the manuscript (include quotes in quotation marks "like this" to indicate direct quotes from your manuscript), or elaborate on this item by providing additional information not in the ms, or briefly explain why the item is not applicable/relevant for your study

"The study team devoted substantial time to troubleshoot unanticipated problems with the eCDSS programming and capture of outcomes. Every update or change to the EHR processes had the potential to disrupt the trial implementation. The team regularly validated the eCDSS and data capture through manual chart reviews. The inaccuracies of EHR data extraction are well-documented [57]. Despite the presence of an explicit trial infrastructure in our EHR, implementation of the intervention and data collection was far from seamless."

4a) Eligibility criteria for participants

Does your paper address CONSORT subitem 4a? *

Copy and paste relevant sections from the manuscript (include quotes in quotation marks "like this" to indicate direct quotes from your manuscript), or elaborate on this item by providing additional information not in the ms, or briefly explain why the item is not applicable/relevant for your study

"All providers practicing at the general internal medicine practice with a primary care panel were eligible for inclusion and randomization.

Eligible patients were identified using a previously described and validated algorithm [40]. (Patients were considered eligible for inclusion if they were aged 18 to 80 years; had a preferred language of English, Spanish, or Chinese; had at least 2 outpatient eGFRCr=30 to 59 milliliters/minute (mL/min) at least 90 days apart, one of which was within the 12 months before August 14, 2017 (when providers were first contacted for study recruitment); and had a primary care visit in the past 18 months. (see Figure 1 for the Consolidated Standards of Reporting Trial diagram) Participants were automatically excluded (based on EHR data) if they were deceased; diagnosed with end-stage renal disease; engaged with a nephrology clinic with 2 or more visits in the past 12 months; kidney transplant recipients; or diagnosed with dementia. PCPs were then sent a list of potential participants for inclusion and asked to exclude participants for additional criteria that were unreliably captured when extracted from the EHR: current pregnancy, life expectancy <6 months, limited communication ability owing to impaired cognition or severe mental illness, New York Heart Association Class III/IV heart failure, or known ejection fraction <25%. PCPs could also exclude any other participants they felt would be inappropriate for study staff to contact.

4a-i) Computer / Internet literacy

Computer / Internet literacy is often an implicit “de facto” eligibility criterion - this should be explicitly clarified.

	1	2	3	4	5	
subitem not at all important	○	○	○	○	○	essential

Does your paper address subitem 4a-i?

Copy and paste relevant sections from the manuscript (include quotes in quotation marks "like this" to indicate direct quotes from your manuscript), or elaborate on this item by providing additional information not in the ms, or briefly explain why the item is not applicable/relevant for your study

No; not relevant as intervention directed at clinicians

4a-ii) Open vs. closed, web-based vs. face-to-face assessments:

Open vs. closed, web-based vs. face-to-face assessments: Mention how participants were recruited (online vs. offline), e.g., from an open access website or from a clinic, and clarify if this was a purely web-based trial, or there were face-to-face components (as part of the intervention or for assessment), i.e., to what degree got the study team to know the participant. In online-only trials, clarify if participants were quasi-anonymous and whether having multiple identities was possible or whether technical or logistical measures (e.g., cookies, email confirmation, phone calls) were used to detect/prevent these.

	1	2	3	4	5	
subitem not at all important	○	○	○	○	○	essential

Does your paper address subitem 4a-ii? *

Copy and paste relevant sections from the manuscript (include quotes in quotation marks "like this" to indicate direct quotes from your manuscript), or elaborate on this item by providing additional information not in the ms, or briefly explain why the item is not applicable/relevant for your study

"We recruited PCPs with eligible patients by eGFRCr by email between August 14, 2017 and September 8, 2017. PCPs were given 2 weeks to either opt-out entirely or exclude individual patients. We then randomized participating PCPs as described below. Participation letters were mailed to eligible intervention arm patients between September 1, 2017 and September 27, 2017, and patients were given 2 weeks to either return an opt-out card or call the study coordinator to opt out. Patients opting out after the study start date of October 4, 2017, were considered withdrawn from the study."

"We utilized block randomization at the PCP level based on panel size. The study statistician randomized PCP participants to each of the 3 study arms using an automated procedure that accounted for cluster size, to assure balance both in terms of number of patients and number of providers per arm. Eligible patient participants were assigned to study arms based on their PCP's assignment. The study statistician was blinded to the identification of the PCPs and patients and will be similarly blinded for the analyses."

4a-iii) Information giving during recruitment

Information given during recruitment. Specify how participants were briefed for recruitment and in the informed consent procedures (e.g., publish the informed consent documentation as appendix, see also item X26), as this information may have an effect on user self-selection, user expectation and may also bias results.

	1	2	3	4	5	
subitem not at all important	○	○	○	○	○	essential

Does your paper address subitem 4a-iii?

Copy and paste relevant sections from the manuscript (include quotes in quotation marks "like this" to indicate direct quotes from your manuscript), or elaborate on this item by providing additional information not in the ms, or briefly explain why the item is not applicable/relevant for your study

"Participation letters were mailed to eligible intervention arm patients between September 1, 2017 and September 27, 2017, and patients were given 2 weeks to either return an opt-out card or call the study coordinator to opt out. Patients opting out after the study start date of October 4, 2017, were considered withdrawn from the study."

4b) Settings and locations where the data were collected

Does your paper address CONSORT subitem 4b? *

Copy and paste relevant sections from the manuscript (include quotes in quotation marks "like this" to indicate direct quotes from your manuscript), or elaborate on this item by providing additional information not in the ms, or briefly explain why the item is not applicable/relevant for your study

"This study was conducted at a general internal medicine practice (with 2 locations) at the University of California San Francisco that cares for a diverse population of more than 24,000 adult patients."

"For patient participants, the EHR was used to collect baseline data about demographic characteristics (age, sex, race-ethnicity, preferred language, and insurance status); medical co-morbidities (cerebrovascular disease, congestive heart failure, coronary artery disease, diabetes mellitus, hyperlipidemia, and hypertension); current medication prescriptions (statin, ACEi/ARB, and diuretics); previous documentation of CKD on problem list and/or as a visit diagnosis, most recent blood pressure, eGFRCr, eGFRCys (if available), and urine ACR (if available) before enrollment.

Primary outcome data on BP will be extracted from the EHR. Baseline BP was defined as the most recent ambulatory BP measurement before final enrollment on October 4, 2017. All patient participant ambulatory BP measurements were collected during the intervention period and will continue to be collected for the 6 months after the end of the intervention.

Secondary outcomes will be collected primarily using automated EHR data extraction algorithms. The outcomes acquired through data extraction include all secondary outcomes except for provider and patient-centered outcomes, which are collected via surveys. Up to 25 participants in each of the 3 arms were surveyed to assess patient knowledge about CKD and NSAID avoidance. Patient participants in the eCDSS arm were surveyed 2 weeks after a visit in which the SmartSet was utilized; those in the eCDSS+ arm were surveyed 2 weeks after their pharmacist follow-up call, and those in the usual care arm were surveyed 2 weeks after a visit with their PCP during the study period. All participating intervention PCPs will be surveyed about their perception of the feasibility and utility of the eCDSS and pharmacist calls (for CDSS+ providers)."

4b-i) Report if outcomes were (self-)assessed through online questionnaires

Clearly report if outcomes were (self-)assessed through online questionnaires (as common in web-based trials) or otherwise.

	1	2	3	4	5	
subitem not at all important	○	○	○	○	○	essential

Does your paper address subitem 4b-i? *

Copy and paste relevant sections from the manuscript (include quotes in quotation marks "like this" to indicate direct quotes from your manuscript), or elaborate on this item by providing additional information not in the ms, or briefly explain why the item is not applicable/relevant for your study

"All participating intervention PCPs will be surveyed about their perception of the feasibility and utility of the eCDSS and pharmacist calls (for CDSS+ providers)."

4b-ii) Report how institutional affiliations are displayed

Report how institutional affiliations are displayed to potential participants [on ehealth media], as affiliations with prestigious hospitals or universities may affect volunteer rates, use, and reactions with regards to an intervention.(Not a required item – describe only if this may bias results)

	1	2	3	4	5	
subitem not at all important	○	○	○	○	○	essential

Does your paper address subitem 4b-ii?

Copy and paste relevant sections from the manuscript (include quotes in quotation marks "like this" to indicate direct quotes from your manuscript), or elaborate on this item by providing additional information not in the ms, or briefly explain why the item is not applicable/relevant for your study

Patients receive care at UCSF so aware of institutional affiliation of study

5) The interventions for each group with sufficient details to allow replication, including how and when they were actually administered

5-i) Mention names, credential, affiliations of the developers, sponsors, and owners

Mention names, credential, affiliations of the developers, sponsors, and owners [6] (if authors/evaluators are owners or developer of the software, this needs to be declared in a “Conflict of interest” section or mentioned elsewhere in the manuscript).

	1	2	3	4	5	
subitem not at all important	○	○	○	○	○	essential

Does your paper address subitem 5-i?

Copy and paste relevant sections from the manuscript (include quotes in quotation marks "like this" to indicate direct quotes from your manuscript), or elaborate on this item by providing additional information not in the ms, or briefly explain why the item is not applicable/relevant for your study

"The EHR used within this practice is Epic (EpicCare, Epic Systems)."

"To optimize the chance of a successful intervention, we worked with a multidisciplinary team (general internist, informatics specialist, and nephrologists) to develop the eCDSS logic. The team focused on areas with the strongest evidence base for CKD management in primary care and developed the logic to apply guideline recommendations in real-time for individual patients."

5-ii) Describe the history/development process

Describe the history/development process of the application and previous formative evaluations (e.g., focus groups, usability testing), as these will have an impact on adoption/use rates and help with interpreting results.

	1	2	3	4	5	
subitem not at all important	○	○	○	○	○	essential

Does your paper address subitem 5-ii?

Copy and paste relevant sections from the manuscript (include quotes in quotation marks "like this" to indicate direct quotes from your manuscript), or elaborate on this item by providing additional information not in the ms, or briefly explain why the item is not applicable/relevant for your study

"To optimize the chance of a successful intervention, we worked with a multidisciplinary team (general internist, informatics specialist, and nephrologists) to develop the eCDSS logic. The team focused on areas with the strongest evidence base for CKD management in primary care and developed the logic to apply guideline recommendations in real-time for individual patients."

"We elicited feedback from PCPs in focus group settings during development of the eCDSS and during implementation planning, which is a recommended practice to increase adoption and uptake of eCDSS [23]. Focus group discussions centered on eCDSS messaging and orders, as well as ways to facilitate integration into the usual clinical workflow."

5-iii) Revisions and updating

Revisions and updating. Clearly mention the date and/or version number of the application/intervention (and comparator, if applicable) evaluated, or describe whether the intervention underwent major changes during the evaluation process, or whether the development and/or content was “frozen” during the trial. Describe dynamic components such as news feeds or changing content which may have an impact on the replicability of the intervention (for unexpected events see item 3b).

	1	2	3	4	5	
subitem not at all important	○	○	○	○	○	essential

Does your paper address subitem 5-iii?

Copy and paste relevant sections from the manuscript (include quotes in quotation marks "like this" to indicate direct quotes from your manuscript), or elaborate on this item by providing additional information not in the ms, or briefly explain why the item is not applicable/relevant for your study

Not relevant; eCDSS created only once

5-iv) Quality assurance methods

Provide information on quality assurance methods to ensure accuracy and quality of information provided [1], if applicable.

	1	2	3	4	5	
subitem not at all important	○	○	○	○	○	essential

Does your paper address subitem 5-iv?

Copy and paste relevant sections from the manuscript (include quotes in quotation marks "like this" to indicate direct quotes from your manuscript), or elaborate on this item by providing additional information not in the ms, or briefly explain why the item is not applicable/relevant for your study

"The study team devoted substantial time to troubleshoot unanticipated problems with the eCDSS programming and capture of outcomes. Every update or change to the EHR processes had the potential to disrupt the trial implementation. The team regularly validated the eCDSS and data capture through manual chart reviews. The inaccuracies of EHR data extraction are well-documented"

5-v) Ensure replicability by publishing the source code, and/or providing screenshots/screen-capture video, and/or providing flowcharts of the algorithms used

Ensure replicability by publishing the source code, and/or providing screenshots/screen-capture video, and/or providing flowcharts of the algorithms used. Replicability (i.e., other researchers should in principle be able to replicate the study) is a hallmark of scientific reporting.

	1	2	3	4	5	
subitem not at all important	○	○	○	○	○	essential

Does your paper address subitem 5-v?

Copy and paste relevant sections from the manuscript (include quotes in quotation marks "like this" to indicate direct quotes from your manuscript), or elaborate on this item by providing additional information not in the ms, or briefly explain why the item is not applicable/relevant for your study

"After the triple-marker testing was ordered, the next time the patient participant visited the lab for their regular clinical care, the triple-marker labs were also collected. We programmed the eCDSS to trigger the first time that the patient participant visited their PCP after all 3 lab results were available. (see Figure 2 for the study workflow) The eCDSS best practice advisory (BPA) appeared above the current medications in the electronic chart (Figures 3 and 4)."

"For patient
participants categorized as high risk, when the PCP accepted the BPA, a SmartSet (EpicCare, Epic Systems) of tailored orders and recommendations appeared (Figure 5)."

"In addition to a reminder of BP targets in CKD, the SmartSet for patient participants classified as high risk for progression and/or complications of CKD used real-time EHR lab and medication data to recommend any of the following that applied:
1. Cardiovascular risk reduction: recommendation for use of statin in all individuals aged ≥50 years not already on a statin, as recommended by Kidney Disease: Improving Global Outcomes (KDIGO) guidelines [17].
2. Potassium management: diet and diuretic recommendations.
3. Proteinuria management: initiation or titration of renin-angiotensin blockade with angiotensin converting enzyme inhibitors (ACEi) or angiotensin II receptor blockers (ARB).
4. Nephrology referral for highest risk participants: highest risk was defined as one of: confirmed eGFRCys <30 mL/min; potassium >5.5 mEq/L; ACR >300 mcg/mg, systolic blood pressure (SBP) >150 millimeters of mercury (mm Hg) despite 3 agents including a diuretic; >3% risk of five-year progression kidney failure based on Tangri equation [41].
5. Patient education: patient education materials to populate in the after-visit summary (AVS) focused on CKD general information, avoidance of nonsteroidal anti-inflammatory drugs (NSAIDs), and dietary recommendations."

5-vi) Digital preservation

Digital preservation: Provide the URL of the application, but as the intervention is likely to change or disappear over the course of the years; also make sure the intervention is archived (Internet Archive, webcitation.org, and/or publishing the source code or screenshots/videos alongside the article). As pages behind login screens cannot be archived, consider creating demo pages which are accessible without login.

	1	2	3	4	5	
subitem not at all important	○	○	○	○	○	essential

Does your paper address subitem 5-vi?

Copy and paste relevant sections from the manuscript (include quotes in quotation marks "like this" to indicate direct quotes from your manuscript), or elaborate on this item by providing additional information not in the ms, or briefly explain why the item is not applicable/relevant for your study

N/A

5-vii) Access

Access: Describe how participants accessed the application, in what setting/context, if they had to pay (or were paid) or not, whether they had to be a member of specific group. If known, describe how participants obtained “access to the platform and Internet” [1]. To ensure access for editors/reviewers/readers, consider to provide a “backdoor” login account or demo mode for reviewers/readers to explore the application (also important for archiving purposes, see vi).

	1	2	3	4	5	
subitem not at all important	○	○	○	○	○	essential

Does your paper address subitem 5-vii? *

Copy and paste relevant sections from the manuscript (include quotes in quotation marks "like this" to indicate direct quotes from your manuscript), or elaborate on this item by providing additional information not in the ms, or briefly explain why the item is not applicable/relevant for your study

Not entirely relevant, as it is an CDSS system embedded in local EHR

"After the triple-marker testing was ordered, the next time the patient participant visited the lab for their regular clinical care, the triple-marker labs were also collected. We programmed the eCDSS to trigger the first time that the patient participant visited their PCP after all 3 lab results were available."

5-viii) Mode of delivery, features/functionalities/components of the intervention and comparator, and the theoretical framework

Describe mode of delivery, features/functionalities/components of the intervention and comparator, and the theoretical framework [6] used to design them (instructional strategy [1], behaviour change techniques, persuasive features, etc., see e.g., [7, 8] for terminology). This includes an in-depth description of the content (including where it is coming from and who developed it) [1],” whether [and how] it is tailored to individual circumstances and allows users to track their progress and receive feedback” [6]. This also includes a description of communication delivery channels and – if computer-mediated communication is a component – whether communication was synchronous or asynchronous [6]. It also includes information on presentation strategies [1], including page design principles, average amount of text on pages, presence of hyperlinks to other resources, etc. [1].

	1	2	3	4	5	
subitem not at all important	○	○	○	○	○	essential

Does your paper address subitem 5-viii? *

Copy and paste relevant sections from the manuscript (include quotes in quotation marks "like this" to indicate direct quotes from your manuscript), or elaborate on this item by providing additional information not in the ms, or briefly explain why the item is not applicable/relevant for your study

"Therefore, we utilized several theory-based strategies to encourage provider uptake of the eCDSS and participant uptake of provider and/or pharmacist recommendations. We classify these strategies using the capability, opportunity, motivation, behavior framework (or COM-B), which asserts that capability, opportunity, and motivation are essential conditions that impact behavior [44,45].

To encourage provider uptake and use of CKD guidelines, we addressed capability barriers (such as knowledge about the guidelines or the eCDSS or forgetting to discuss CKD owing to limited bandwidth). During the recruitment phase, we educated eligible PCPs about the prevalence of CKD within their panel by sending a list of their potentially eligible participants along with information about the importance of recognizing and managing CKD early. Shortly before

intervention implementation, we sent a training video about the CKD guidelines and the eCDSS to the participating intervention PCPs. The eCDSS itself also served as a prompt to discuss CKD during the visits.

To address PCP opportunity barriers, we designed the eCDSS to fit into the physician workflow and to contain all the necessary CKD-related orders and patient education in one easily accessible place during a clinical visit.

To address PCP motivation barriers, we provided a small incentive to the participating intervention PCPs who watched the training video. Finally, to enhance use of the BPA, study personnel sent an individual email reminder to the intervention PCP the day before each eligible patient participant visit.

Although the eCDSS itself was a provider-facing intervention, we used strategies to encourage patient participant behavior change. To address capability barriers (such as knowledge of CKD or forgetting the provider recommendations), the CDSS SmartSet had CKD-based patient educational handouts in the patient’s preferred language. The pharmacist phone call was also designed to reinforce the knowledge provided at the office visit and motivate the patient participant to adhere to medication and avoid NSAIDS."

5-ix) Describe use parameters

Describe use parameters (e.g., intended “doses” and optimal timing for use). Clarify what instructions or recommendations were given to the user, e.g., regarding timing, frequency, heaviness of use, if any, or was the intervention used ad libitum.

	1	2	3	4	5	
subitem not at all important	○	○	○	○	○	essential

Does your paper address subitem 5-ix?

Copy and paste relevant sections from the manuscript (include quotes in quotation marks "like this" to indicate direct quotes from your manuscript), or elaborate on this item by providing additional information not in the ms, or briefly explain why the item is not applicable/relevant for your study

See Figure 3

5-x) Clarify the level of human involvement

Clarify the level of human involvement (care providers or health professionals, also technical assistance) in the e-intervention or as co-intervention (detail number and expertise of professionals involved, if any, as well as “type of assistance offered, the timing and frequency of the support, how it is initiated, and the medium by which the assistance is delivered”. It may be necessary to distinguish between the level of human involvement required for the trial, and the level of human involvement required for a routine application outside of a RCT setting (discuss under item 21 – generalizability).

	1	2	3	4	5	
subitem not at all important	○	○	○	○	○	essential

Does your paper address subitem 5-x?

Copy and paste relevant sections from the manuscript (include quotes in quotation marks "like this" to indicate direct quotes from your manuscript), or elaborate on this item by providing additional information not in the ms, or briefly explain why the item is not applicable/relevant for your study

"To optimize the chance of a successful intervention, we worked with a multidisciplinary team (general internist, informatics specialist, and nephrologists) to develop the eCDSS logic."

"The second intervention arm (eCDSS+) included a pharmacist follow-up visit by telephone. If the SmartSet was signed during the PCP visit, a pharmacist would call the participant within 2 weeks of the visit to reinforce CKD-related teaching and medication changes from the visit as well as complete a comprehensive medication review. Information on the telephone encounter was documented in the EHR and sent to the PCP; any urgent clinical issues identified during the call were highlighted for the PCP as needing their follow-up. Providers in the eCDSS+ group also were encouraged to make a warm handoff to the pharmacist by including anticipatory guidance about the pharmacist phone call within the after-visit summary and distributing a business card with the pharmacist’s photo and phone number for their patient participants at the time of using the SmartSet.
Given that the study physician ordered labs at the start of the intervention period, safety checks were put into place to ensure adequate review of the laboratory results. A nephrologist (LL) from the study team reviewed weekly laboratory results specifically to identify the following: eGFRCr decline >30% from baseline, ACR ≥1000 mcg/mg, adherence to nephrology referrals, and any discordance >30% between eGFRCr and eGFRCys. In all of these situations, the nephrologist contacted the PCP to ensure appropriate follow-up."

5-xi) Report any prompts/reminders used

Report any prompts/reminders used: Clarify if there were prompts (letters, emails, phone calls, SMS) to use the application, what triggered them, frequency etc. It may be necessary to distinguish between the level of prompts/reminders required for the trial, and the level of prompts/reminders for a routine application outside of a RCT setting (discuss under item 21 – generalizability).

	1	2	3	4	5	
subitem not at all important	○	○	○	○	○	essential

Does your paper address subitem 5-xi? *

Copy and paste relevant sections from the manuscript (include quotes in quotation marks "like this" to indicate direct quotes from your manuscript), or elaborate on this item by providing additional information not in the ms, or briefly explain why the item is not applicable/relevant for your study

See figure 3. "We programmed the
eCDSS to trigger the first time that the patient participant visited
their PCP after all 3 lab results were available. (see Figure 2 for
the study workflow) The eCDSS best practice advisory (BPA)
appeared above the current medications in the electronic chart"

"If the PCP did not
open the BPA and sign the orders in the SmartSet during the
first PCP visit after the triple-marker labs were available, the
BPA would trigger at up to 2 additional PCP visits during the
study period. The eCDSS did not trigger if the patient participant
saw a provider who was not their PCP."

"Finally, to enhance use of the BPA, study
personnel sent an individual email reminder to the intervention
PCP the day before each eligible patient participant visit."

5-xii) Describe any co-interventions (incl. training/support)

Describe any co-interventions (incl. training/support): Clearly state any interventions that are provided in addition to the targeted eHealth intervention, as ehealth intervention may not be designed as stand-alone intervention. This includes training sessions and support [1]. It may be necessary to distinguish between the level of training required for the trial, and the level of training for a routine application outside of a RCT setting (discuss under item 21 – generalizability.

	1	2	3	4	5	
subitem not at all important	○	○	○	○	○	essential

Does your paper address subitem 5-xii? *

Copy and paste relevant sections from the manuscript (include quotes in quotation marks "like this" to indicate direct quotes from your manuscript), or elaborate on this item by providing additional information not in the ms, or briefly explain why the item is not applicable/relevant for your study

Yes, pharmacy support. ""The second intervention arm (eCDSS+) included a pharmacist follow-up visit by telephone. If the SmartSet was signed during the PCP visit, a pharmacist would call the participant within 2 weeks of the visit to reinforce CKD-related teaching and medication changes from the visit as well as complete a comprehensive medication review. Information on the telephone encounter was documented in the EHR and sent to the PCP; any urgent clinical issues identified during the call were highlighted for the PCP as needing their follow-up. Providers in the eCDSS+ group also were encouraged to make a warm handoff to the pharmacist by including anticipatory guidance about the pharmacist phone call within the after-visit summary and distributing a business card with the pharmacist’s photo and phone number for their patient participants at the time of using the SmartSet."

6a) Completely defined pre-specified primary and secondary outcome measures, including how and when they were assessed

Does your paper address CONSORT subitem 6a? *

Copy and paste relevant sections from the manuscript (include quotes in quotation marks "like this" to indicate direct quotes from your manuscript), or elaborate on this item by providing additional information not in the ms, or briefly explain why the item is not applicable/relevant for your study

See figure 6 (outcomes)

"Primary Outcome
A summary of pre-planned study outcomes is shown in Figure 6. The primary clinical outcome of this study is BP change from baseline. This outcome will be assessed as a continuous variable (separately for both diastolic and systolic BP change from baseline) and dichotomous variable (sustained control, defined as BP <140/90 in 2 or more consecutive visits during the trial). As this study was initiated before the 2017 BP guidelines, <140/90 was used to define control; this value also aligns with current KDIGO recommendations [17,46]. Rates of control at the end of the intervention period and 6 months later will be assessed based on the most recent BP measurement carried forward.
Secondary Outcomes
Our main secondary outcome is PCP awareness of the patient participant's CKD diagnosis, as measured by inclusion of CKD-related International Statistical Classification of Diseases and Related Health Problems-10th Revision codes on the problem list or as a visit diagnosis. Additional secondary outcomes include process of care, patient-centered, and implementation outcomes.

There are 2 main process of care outcomes: (1) the proportion of patient participants with albuminuria prescribed ACEi/ARB and (2) appropriate use of statins. Appropriate statin use is defined both as the proportion of all patient participants aged ≥50 years prescribed statin therapy and the proportion of participants initiated on statin therapy after study start date. Patient-centered outcomes include patient knowledge about CKD and NSAID avoidance as measured by patient surveys.
In addition to the effectiveness outcomes described above, the implementation outcomes are described in detail in Figure 6 using the reach, effectiveness, adoption, implementation, and maintenance (or RE-AIM) framework to highlight measures [illegible]

crucial to successful implementation [47]. In this paper, we report initial implementation results, including all of the Reach outcomes and the first Adoption outcome (PCP SmartSet use)."

"For patient participants, the EHR was used to collect baseline data about demographic characteristics (age, sex, race-ethnicity, preferred language, and insurance status); medical co-morbidities (cerebrovascular disease, congestive heart failure, coronary artery disease, diabetes mellitus, hyperlipidemia, and hypertension); current medication prescriptions (statin, ACEi/ARB, and diuretics); previous documentation of CKD on problem list and/or as a visit diagnosis, most recent blood pressure, eGFRCr, eGFRCys (if available), and urine ACR (if available) before enrollment.
Primary outcome data on BP will be extracted from the EHR. Baseline BP was defined as the most recent ambulatory BP measurement before final enrollment on October 4, 2017. All patient participant ambulatory BP measurements were collected during the intervention period and will continue to be collected for the 6 months after the end of the intervention.
Secondary outcomes will be collected primarily using automated EHR data extraction algorithms. The outcomes acquired through data extraction include all secondary outcomes except for provider and patient-centered outcomes, which are collected via surveys. Up to 25 participants in each of the 3 arms were surveyed to assess patient knowledge about CKD and NSAID avoidance. Patient participants in the eCDSS arm were surveyed 2 weeks after a visit in which the SmartSet was utilized; those in the eCDSS+ arm were surveyed 2 weeks after their pharmacist follow-up call, and those in the usual care arm were surveyed 2 weeks after a visit with their PCP during the study period. All participating intervention PCPs will be surveyed about their perception of the feasibility and utility of the eCDSS and pharmacist calls (for CDSS+ providers)."

6a-i) Online questionnaires: describe if they were validated for online use and apply CHERRIES items to describe how the questionnaires were designed/deployed

If outcomes were obtained through online questionnaires, describe if they were validated for online use and apply CHERRIES items to describe how the questionnaires were designed/deployed [9].

	1	2	3	4	5	
subitem not at all important	○	○	○	○	○	essential

Does your paper address subitem 6a-i?

Copy and paste relevant sections from manuscript text

N/A: no online questionnaires currently

6a-ii) Describe whether and how “use” (including intensity of use/dosage) was defined/measured/monitored

Describe whether and how “use” (including intensity of use/dosage) was defined/measured/monitored (logins, logfile analysis, etc.). Use/adoption metrics are important process outcomes that should be reported in any ehealth trial.

	1	2	3	4	5	
subitem not at all important	○	○	○	○	○	essential

Does your paper address subitem 6a-ii?

Copy and paste relevant sections from manuscript text

See figure 6: implementation outcomes

"In addition to the effectiveness outcomes described above, the implementation outcomes are described in detail in Figure 6 using the reach, effectiveness, adoption, implementation, and maintenance (or RE-AIM) framework to highlight measures crucial to successful implementation [47]. In this paper, we report initial implementation results, including all of the Reach outcomes and the first Adoption outcome (PCP SmartSet use)."

6a-iii) Describe whether, how, and when qualitative feedback from participants was obtained

Describe whether, how, and when qualitative feedback from participants was obtained (e.g., through emails, feedback forms, interviews, focus groups).

	1	2	3	4	5	
subitem not at all important	○	○	○	○	○	essential

Does your paper address subitem 6a-iii?

Copy and paste relevant sections from manuscript text

N/A: protocol paper. Planned qualitative data collection reported.

"Patient participants in the eCDSS arm were surveyed 2 weeks after a visit in which the SmartSet was utilized; those in the eCDSS+ arm were surveyed 2 weeks after their pharmacist follow-up call, and those in the usual care arm were surveyed 2 weeks after a visit with their PCP during the study period. All participating intervention PCPs will be surveyed about their perception of the feasibility and utility of the eCDSS and pharmacist calls (for CDSS+ providers)."

6b) Any changes to trial outcomes after the trial commenced, with reasons

Does your paper address CONSORT subitem 6b? *

Copy and paste relevant sections from the manuscript (include quotes in quotation marks "like this" to indicate direct quotes from your manuscript), or elaborate on this item by providing additional information not in the ms, or briefly explain why the item is not applicable/relevant for your study

No

7a) How sample size was determined

NPT: When applicable, details of whether and how the clustering by care provides or centers was addressed

7a-i) Describe whether and how expected attrition was taken into account when calculating the sample size

Describe whether and how expected attrition was taken into account when calculating the sample size.

	1	2	3	4	5	
subitem not at all important	○	○	○	○	○	essential

Does your paper address subitem 7a-i?

Copy and paste relevant sections from manuscript title (include quotes in quotation marks "like this" to indicate direct quotes from your manuscript), or elaborate on this item by providing additional information not in the ms, or briefly explain why the item is not applicable/relevant for your study

Based on preliminary data from our institution's EHR, it was determined that there would be a maximum of 1400 participants in the practice who could meet inclusion criteria. The study was powered for the clinical outcome of BP change, with calculations performed using the clustersampsi command for Stata version 11.2. We assumed a 2-tailed alpha level of 0.05, an intraclass correlation coefficient of 0.025 based on studies in primary care settings [49,50], and a standard deviation of 5 mmHg. Assuming 23 clinicians per arm with at least 15 patients per clinician (ie, 345 patients per arm), we estimated that we would have 80% power to detect a difference as small as 1.27 mmHg between arms.

7b) When applicable, explanation of any interim analyses and stopping guidelines

Does your paper address CONSORT subitem 7b? *

Copy and paste relevant sections from the manuscript (include quotes in quotation marks "like this" to indicate direct quotes from your manuscript), or elaborate on this item by providing additional information not in the ms, or briefly explain why the item is not applicable/relevant for your study

N/A

8a) Method used to generate the random allocation sequence

NPT: When applicable, how care providers were allocated to each trial group

Does your paper address CONSORT subitem 8a? *

Copy and paste relevant sections from the manuscript (include quotes in quotation marks "like this" to indicate direct quotes from your manuscript), or elaborate on this item by providing additional information not in the ms, or briefly explain why the item is not applicable/relevant for your study

"We utilized block randomization at the PCP level based on panel size. The study statistician randomized PCP participants to each of the 3 study arms using an automated procedure that accounted for cluster size, to assure balance both in terms of number of patients and number of providers per arm. Eligible patient participants were assigned to study arms based on their PCP's assignment."

8b) Type of randomisation; details of any restriction (such as blocking and block size)

Does your paper address CONSORT subitem 8b? *

Copy and paste relevant sections from the manuscript (include quotes in quotation marks "like this" to indicate direct quotes from your manuscript), or elaborate on this item by providing additional information not in the ms, or briefly explain why the item is not applicable/relevant for your study

"We utilized block randomization at the PCP level based on panel size. The study statistician randomized PCP participants to each of the 3 study arms using an automated procedure that accounted for cluster size, to assure balance both in terms of number of patients and number of providers per arm. Eligible patient participants were assigned to study arms based on their PCP's assignment."

9) Mechanism used to implement the random allocation sequence (such as sequentially numbered containers), describing any steps taken to conceal the sequence until interventions were assigned

Does your paper address CONSORT subitem 9? *

Copy and paste relevant sections from the manuscript (include quotes in quotation marks "like this" to indicate direct quotes from your manuscript), or elaborate on this item by providing additional information not in the ms, or briefly explain why the item is not applicable/relevant for your study

"We utilized block randomization at the PCP level based on panel size. The study statistician randomized PCP participants to each of the 3 study arms using an automated procedure that accounted for cluster size, to assure balance both in terms of number of patients and number of providers per arm. Eligible patient participants were assigned to study arms based on their PCP's assignment."

10) Who generated the random allocation sequence, who enrolled participants, and who assigned participants to interventions

Does your paper address CONSORT subitem 10? *

Copy and paste relevant sections from the manuscript (include quotes in quotation marks "like this" to indicate direct quotes from your manuscript), or elaborate on this item by providing additional information not in the ms, or briefly explain why the item is not applicable/relevant for your study

"We utilized block randomization at the PCP level based on panel size. The study statistician randomized PCP participants to each of the 3 study arms using an automated procedure that accounted for cluster size, to assure balance both in terms of number of patients and number of providers per arm. Eligible patient participants were assigned to study arms based on their PCP's assignment."

11a) If done, who was blinded after assignment to interventions (for example, participants, care providers, those assessing outcomes) and how

NPT: Whether or not administering co-interventions were blinded to group assignment

11a-i) Specify who was blinded, and who wasn't

Specify who was blinded, and who wasn't. Usually, in web-based trials it is not possible to blind the participants [1, 3] (this should be clearly acknowledged), but it may be possible to blind outcome assessors, those doing data analysis or those administering co-interventions (if any).

	1	2	3	4	5	
subitem not at all important	○	○	○	○	○	essential

Does your paper address subitem 11a-i? *

Copy and paste relevant sections from the manuscript (include quotes in quotation marks "like this" to indicate direct quotes from your manuscript), or elaborate on this item by providing additional information not in the ms, or briefly explain why the item is not applicable/relevant for your study

"The study statistician was blinded to the identification of the PCPs and patients and will be similarly blinded for the analyses."

Care providers and patients were not blinded.

11a-ii) Discuss e.g., whether participants knew which intervention was the "intervention of interest" and which one was the "comparator"

Informed consent procedures (4a-ii) can create biases and certain expectations - discuss e.g., whether participants knew which intervention was the "intervention of interest" and which one was the "comparator".

	1	2	3	4	5	
subitem not at all important	○	○	○	○	○	essential

Does your paper address subitem 11a-ii?

Copy and paste relevant sections from the manuscript (include quotes in quotation marks "like this" to indicate direct quotes from your manuscript), or elaborate on this item by providing additional information not in the ms, or briefly explain why the item is not applicable/relevant for your study

Not discussed in detail. PCPs are aware if they receive the eCDSS with or without pharmacist phone call. Patients only knew they were enrolled in the study and would notice if a pharmacist called them.

"Participation letters were mailed to eligible intervention arm patients between September 1, 2017 and September 27, 2017, and patients were given 2 weeks to either return an opt-out card or call the study coordinator to opt out. Patients opting out after the study start date of October 4, 2017, were considered withdrawn from the study.
The usual care group PCPs received no additional contact beyond recruitment from the study team, and the usual care patients were never contacted directly by the study team. Usual care PCPs did have access to the same labs and medications to use at their clinical discretion without any recommendations or direction from the study team."

11b) If relevant, description of the similarity of interventions

(this item is usually not relevant for ehealth trials as it refers to similarity of a placebo or sham intervention to a active medication/intervention)

Does your paper address CONSORT subitem 11b? *

Copy and paste relevant sections from the manuscript (include quotes in quotation marks "like this" to indicate direct quotes from your manuscript), or elaborate on this item by providing additional information not in the ms, or briefly explain why the item is not applicable/relevant for your study

Usual care received no other contact.

12a) Statistical methods used to compare groups for primary and secondary outcomes

NPT: When applicable, details of whether and how the clustering by care providers or centers was addressed

Does your paper address CONSORT subitem 12a? *

Copy and paste relevant sections from the manuscript (include quotes in quotation marks "like this" to indicate direct quotes from your manuscript), or elaborate on this item by providing additional information not in the ms, or briefly explain why the item is not applicable/relevant for your study

This is a protocol paper that reports planned analyses: "Baseline demographic and clinical characteristics will be
summarized and study balance by study arm will be assessed
using descriptive statistics as well as methods that account for
the lack of independence among patients in the same cluster
[48]. Balance will be assessed with logit link generalized
estimating equation (GEE) for categorical variables and linear
GEE models for continuous variables. Clustering will be
accounted for using an exchangeable correlation matrix with
robust standard errors. We anticipate controlling for
characteristics that are associated with outcomes but differ at
baseline between arms and using multivariable regression
analyses with GEE to account for cluster randomization in the
reporting of the final outcomes of our study. Sensitivity analyses
will include as treated analyses with inverse probability
weighting to determine impact of participants changing
providers/study arms as well as to determine impact of any
differences in the number of assessments or clinic visits by study
arm. Primary analyses will follow intention-to-treat principles."

12a-i) Imputation techniques to deal with attrition / missing values

Imputation techniques to deal with attrition / missing values: Not all participants will use the intervention/comparator as intended and attrition is typically high in ehealth trials. Specify how participants who did not use the application or dropped out from the trial were treated in the statistical analysis (a complete case analysis is strongly discouraged, and simple imputation techniques such as LOCF may also be problematic [4]).

	1	2	3	4	5	
subitem not at all important	○	○	○	○	○	essential

Does your paper address subitem 12a-i? *

Copy and paste relevant sections from the manuscript (include quotes in quotation marks "like this" to indicate direct quotes from your manuscript), or elaborate on this item by providing additional information not in the ms, or briefly explain why the item is not applicable/relevant for your study

N/A: protocol paper

12b) Methods for additional analyses, such as subgroup analyses and adjusted analyses

Does your paper address CONSORT subitem 12b? *

Copy and paste relevant sections from the manuscript (include quotes in quotation marks "like this" to indicate direct quotes from your manuscript), or elaborate on this item by providing additional information not in the ms, or briefly explain why the item is not applicable/relevant for your study

"Baseline demographic and clinical characteristics will be summarized and study balance by study arm will be assessed using descriptive statistics as well as methods that account for the lack of independence among patients in the same cluster [48]. Balance will be assessed with logit link generalized estimating equation (GEE) for categorical variables and linear GEE models for continuous variables. Clustering will be accounted for using an exchangeable correlation matrix with robust standard errors. We anticipate controlling for characteristics that are associated with outcomes but differ at baseline between arms and using multivariable regression analyses with GEE to account for cluster randomization in the reporting of the final outcomes of our study. Sensitivity analyses will include as treated analyses with inverse probability weighting to determine impact of participants changing providers/study arms as well as to determine impact of any differences in the number of assessments or clinic visits by study arm. Primary analyses will follow intention-to-treat principles."

X26) REB/IRB Approval and Ethical Considerations [recommended as subheading under "Methods"] (not a CONSORT item)

X26-i) Comment on ethics committee approval

	1	2	3	4	5	
subitem not at all important	○	○	○	○	○	essential

Does your paper address subitem X26-i?

Copy and paste relevant sections from the manuscript (include quotes in quotation marks "like this" to indicate direct quotes from your manuscript), or elaborate on this item by providing additional information not in the ms, or briefly explain why the item is not applicable/relevant for your study

"The University of California San Francisco Human Research Protection Program approved the protocol for this study."

x26-ii) Outline informed consent procedures

Outline informed consent procedures e.g., if consent was obtained offline or online (how? Checkbox, etc.?), and what information was provided (see 4a-ii). See [6] for some items to be included in informed consent documents.

	1	2	3	4	5	
subitem not at all important	○	○	○	○	○	essential

Does your paper address subitem X26-ii?

Copy and paste relevant sections from the manuscript (include quotes in quotation marks "like this" to indicate direct quotes from your manuscript), or elaborate on this item by providing additional information not in the ms, or briefly explain why the item is not applicable/relevant for your study

"We recruited PCPs with eligible patients by eGFRCr by email between August 14, 2017 and September 8, 2017. PCPs were given 2 weeks to either opt-out entirely or exclude individual patients. We then randomized participating PCPs as described below. Participation letters were mailed to eligible intervention arm patients between September 1, 2017 and September 27, 2017, and patients were given 2 weeks to either return an opt-out card or call the study coordinator to opt out. Patients opting out after the study start date of October 4, 2017, were considered withdrawn from the study.
The usual care group PCPs received no additional contact beyond recruitment from the study team, and the usual care patients were never contacted directly by the study team"

X26-iii) Safety and security procedures

Safety and security procedures, incl. privacy considerations, and any steps taken to reduce the likelihood or detection of harm (e.g., education and training, availability of a hotline)

	1	2	3	4	5	
subitem not at all important	○	○	○	○	○	essential

Does your paper address subitem X26-iii?

Copy and paste relevant sections from the manuscript (include quotes in quotation marks "like this" to indicate direct quotes from your manuscript), or elaborate on this item by providing additional information not in the ms, or briefly explain why the item is not applicable/relevant for your study

"Given that the study physician ordered labs at the start of the intervention period, safety checks were put into place to ensure adequate review of the laboratory results. A nephrologist (LL) from the study team reviewed weekly laboratory results specifically to identify the following: eGFRCr decline >30% from baseline, ACR ≥1000 mcg/mg, adherence to nephrology referrals, and any discordance >30% between eGFRCr and eGFRCys. In all of these situations, the nephrologist contacted the PCP to ensure appropriate follow-up. Specifically, in cases of discordance between the 2 types of eGFR levels, the nephrologist advised PCPs to dose medications based on eGFRCys when the clinical scenario suggested that eGFRcr may not be accurate."

RESULTS

13a) For each group, the numbers of participants who were randomly assigned, received intended treatment, and were analysed for the primary outcome

NPT: The number of care providers or centers performing the intervention in each group and the number of patients treated by each care provider in each center

Does your paper address CONSORT subitem 13a? *

Copy and paste relevant sections from the manuscript (include quotes in quotation marks "like this" to indicate direct quotes from your manuscript), or elaborate on this item by providing additional information not in the ms, or briefly explain why the item is not applicable/relevant for your study

Not fully applicable: protocol paper.

"Recruitment Rate
At the provider level, all 81 eligible PCPs (47 faculty attending physicians, 31 resident physicians, and 3 NPs) agreed to participate (100%). In total, 79 of the 81 providers (98%) were included because 2 providers had no remaining eligible participants after exclusion and opt-outs. At the patient level, 995 patients who met the initial eligibility criteria were identified. A total of 316 / 995 (31.8%) potential patient participants were excluded using automated algorithms, and clinicians excluded another 90 patients (Figure 1). A total of 582 patient participants were distributed to the 3 arms based on provider randomization. After an additional 3 patients were excluded (owing to patient deaths not recorded in EHR before randomization) and 55 / 582 patients (9.5%) opted out or withdrew, 524 patient participants (90.0% of eligible 582 participants) were included in the study: 188 usual care, 165 eCDSS, and 171 eCDSS+.
Baseline patient participant characteristics are shown in Table 1. The 55 patients that opted out/withdrew were similar to the patient participants across multiple traits—including age, gender, race/ethnicity, language preference, and co-morbidities—except they had lower renal function on average: eGFRCr (52.7 [SD 11.7] versus 56.0 [SD 11.8]; P=.046).
Completion of Triple Marker
Of the 336 patient participants in the intervention arms, 178 (53.0%) completed the triple-marker screening.
PCP Visits
Of the 178 patient participants who completed a triple-marker screen, 138 (77.5%) had a PCP visit during the 12-month intervention period. This was 41.1% (138/336) of all intervention participants.
PCP BPA Use
Of the 138 participants with a PCP visit, the BPA was opened for 102 participants (73.9%). This was 30.3% (102/336) of all intervention participants

intervention participants.
Implementation Outcomes: Adoption
SmartSet Use: Of the 102 participants in which the BPA was opened, orders were signed or patient education"

13b) For each group, losses and exclusions after randomisation, together with reasons

Does your paper address CONSORT subitem 13b? (NOTE: Preferably, this is shown in a CONSORT flow diagram) *

Copy and paste relevant sections from the manuscript (include quotes in quotation marks "like this" to indicate direct quotes from your manuscript), or elaborate on this item by providing additional information not in the ms, or briefly explain why the item is not applicable/relevant for your study

Also see Figure 1: consort diagram

""Recruitment Rate
At the provider level, all 81 eligible PCPs (47 faculty attending physicians, 31 resident physicians, and 3 NPs) agreed to participate (100%). In total, 79 of the 81 providers (98%) were included because 2 providers had no remaining eligible participants after exclusion and opt-outs. At the patient level, 995 patients who met the initial eligibility criteria were identified. A total of 316 / 995 (31.8%) potential patient participants were excluded using automated algorithms, and clinicians excluded another 90 patients (Figure 1). A total of 582 patient participants were distributed to the 3 arms based on provider randomization. After an additional 3 patients were excluded (owing to patient deaths not recorded in EHR before randomization) and 55 / 582 patients (9.5%) opted out or withdrew, 524 patient participants (90.0% of eligible 582 participants) were included in the study: 188 usual care, 165 eCDSS, and 171 eCDSS+.
Baseline patient participant characteristics are shown in Table 1. The 55 patients that opted out/withdrew were similar to the patient participants across multiple traits—including age, gender, race/ethnicity, language preference, and co-morbidities—except they had lower renal function on average: eGFRCr (52.7 [SD 11.7] versus 56.0 [SD 11.8]; P=.046)."

13b-i) Attrition diagram

Strongly recommended: An attrition diagram (e.g., proportion of participants still logging in or using the intervention/comparator in each group plotted over time, similar to a survival curve) or other figures or tables demonstrating usage/dose/engagement.

	1	2	3	4	5	
subitem not at all important	○	○	○	○	○	essential

Does your paper address subitem 13b-i?

Copy and paste relevant sections from the manuscript or cite the figure number if applicable (include quotes in quotation marks "like this" to indicate direct quotes from your manuscript), or elaborate on this item by providing additional information not in the ms, or briefly explain why the item is not applicable/relevant for your study

Not fully applicable. Protocol paper with early implementation outcomes.

"Completion of Triple Marker
Of the 336 patient participants in the intervention arms, 178 (53.0%) completed the triple-marker screening.
PCP Visits
Of the 178 patient participants who completed a triple-marker screen, 138 (77.5%) had a PCP visit during the 12-month intervention period. This was 41.1% (138/336) of all intervention participants.
PCP BPA Use
Of the 138 participants with a PCP visit, the BPA was opened for 102 participants (73.9%). This was 30.3% (102/336) of all intervention participants.
Implementation Outcomes: Adoption
SmartSet Use: Of the 102 participants in which the BPA was opened, orders were signed or patient education was given from the SmartSet for 83 participants (81.4%)."

14a) Dates defining the periods of recruitment and follow-up

Does your paper address CONSORT subitem 14a? *

Copy and paste relevant sections from the manuscript (include quotes in quotation marks "like this" to indicate direct quotes from your manuscript), or elaborate on this item by providing additional information not in the ms, or briefly explain why the item is not applicable/relevant for your study

"We planned for an 18-month trial beginning October 4, 2017, that included a 12-month intervention period and subsequent 6-month follow-up period."

"We recruited PCPs with eligible patients by eGFRCr by email between August 14, 2017 and September 8, 2017. PCPs were given 2 weeks to either opt-out entirely or exclude individual patients. We then randomized participating PCPs as described below. Participation letters were mailed to eligible intervention arm patients between September 1, 2017 and September 27, 2017, and patients were given 2 weeks to either return an opt-out card or call the study coordinator to opt out. Patients opting out after the study start date of October 4, 2017, were considered withdrawn from the study."

14a-i) Indicate if critical “secular events” fell into the study period

Indicate if critical “secular events” fell into the study period, e.g., significant changes in Internet resources available or “changes in computer hardware or Internet delivery resources”

	1	2	3	4	5	
subitem not at all important	○	○	○	○	○	essential

Does your paper address subitem 14a-i?

Copy and paste relevant sections from the manuscript (include quotes in quotation marks "like this" to indicate direct quotes from your manuscript), or elaborate on this item by providing additional information not in the ms, or briefly explain why the item is not applicable/relevant for your study

N/A

14b) Why the trial ended or was stopped (early)

Does your paper address CONSORT subitem 14b? *

Copy and paste relevant sections from the manuscript (include quotes in quotation marks "like this" to indicate direct quotes from your manuscript), or elaborate on this item by providing additional information not in the ms, or briefly explain why the item is not applicable/relevant for your study

N/A: trial did not end or stop early

15) A table showing baseline demographic and clinical characteristics for each group

NPT: When applicable, a description of care providers (case volume, qualification, expertise, etc.) and centers (volume) in each group

Does your paper address CONSORT subitem 15? *

Copy and paste relevant sections from the manuscript (include quotes in quotation marks "like this" to indicate direct quotes from your manuscript), or elaborate on this item by providing additional information not in the ms, or briefly explain why the item is not applicable/relevant for your study

See Table 1

15-i) Report demographics associated with digital divide issues

In ehealth trials it is particularly important to report demographics associated with digital divide issues, such as age, education, gender, social-economic status, computer/Internet/ehealth literacy of the participants, if known.

	1	2	3	4	5	
subitem not at all important	○	○	○	○	○	essential

Does your paper address subitem 15-i? *

Copy and paste relevant sections from the manuscript (include quotes in quotation marks "like this" to indicate direct quotes from your manuscript), or elaborate on this item by providing additional information not in the ms, or briefly explain why the item is not applicable/relevant for your study

See Table 1: age, gender, r/e, language, insurance type reported

16) For each group, number of participants (denominator) included in each analysis and whether the analysis was by original assigned groups

16-i) Report multiple “denominators” and provide definitions

Report multiple “denominators” and provide definitions: Report N’s (and effect sizes) “across a range of study participation [and use] thresholds” [1], e.g., N exposed, N consented, N used more than x times, N used more than y weeks, N participants “used” the intervention/comparator at specific pre-defined time points of interest (in absolute and relative numbers per group). Always clearly define “use” of the intervention.

	1	2	3	4	5	
subitem not at all important	○	○	○	○	○	essential

Does your paper address subitem 16-i? *

Copy and paste relevant sections from the manuscript (include quotes in quotation marks "like this" to indicate direct quotes from your manuscript), or elaborate on this item by providing additional information not in the ms, or briefly explain why the item is not applicable/relevant for your study

See figure 1 (consort diagram). Data collection not finalized. This is a protocol paper

16-ii) Primary analysis should be intent-to-treat

Primary analysis should be intent-to-treat, secondary analyses could include comparing only “users”, with the appropriate caveats that this is no longer a randomized sample (see 18-i).

	1	2	3	4	5	
subitem not at all important	○	○	○	○	○	essential

Does your paper address subitem 16-ii?

Copy and paste relevant sections from the manuscript (include quotes in quotation marks "like this" to indicate direct quotes from your manuscript), or elaborate on this item by providing additional information not in the ms, or briefly explain why the item is not applicable/relevant for your study

"Baseline demographic and clinical characteristics will be summarized and study balance by study arm will be assessed using descriptive statistics as well as methods that account for the lack of independence among patients in the same cluster [48]. Balance will be assessed with logit link generalized estimating equation (GEE) for categorical variables and linear GEE models for continuous variables. Clustering will be accounted for using an exchangeable correlation matrix with robust standard errors. We anticipate controlling for characteristics that are associated with outcomes but differ at baseline between arms and using multivariable regression analyses with GEE to account for cluster randomization in the reporting of the final outcomes of our study. Sensitivity analyses will include as treated analyses with inverse probability weighting to determine impact of participants changing providers/study arms as well as to determine impact of any differences in the number of assessments or clinic visits by study arm. Primary analyses will follow intention-to-treat principles."

17a) For each primary and secondary outcome, results for each group, and the estimated effect size and its precision (such as 95% confidence interval)

Does your paper address CONSORT subitem 17a? *

Copy and paste relevant sections from the manuscript (include quotes in quotation marks "like this" to indicate direct quotes from your manuscript), or elaborate on this item by providing additional information not in the ms, or briefly explain why the item is not applicable/relevant for your study

Not applicable: protocol paper

17a-i) Presentation of process outcomes such as metrics of use and intensity of use

In addition to primary/secondary (clinical) outcomes, the presentation of process outcomes such as metrics of use and intensity of use (dose, exposure) and their operational definitions is critical. This does not only refer to metrics of attrition (13-b) (often a binary variable), but also to more continuous exposure metrics such as “average session length”. These must be accompanied by a technical description how a metric like a “session” is defined (e.g., timeout after idle time) [1] (report under item 6a).

	1	2	3	4	5	
subitem not at all important	○	○	○	○	○	essential

Does your paper address subitem 17a-i?

Copy and paste relevant sections from the manuscript (include quotes in quotation marks "like this" to indicate direct quotes from your manuscript), or elaborate on this item by providing additional information not in the ms, or briefly explain why the item is not applicable/relevant for your study

Not fully applicable: protocol paper. Have some interim use metrics

"Completion of Triple Marker
Of the 336 patient participants in the intervention arms, 178 (53.0%) completed the triple-marker screening.
PCP Visits
Of the 178 patient participants who completed a triple-marker screen, 138 (77.5%) had a PCP visit during the 12-month intervention period. This was 41.1% (138/336) of all intervention participants.
PCP BPA Use
Of the 138 participants with a PCP visit, the BPA was opened for 102 participants (73.9%). This was 30.3% (102/336) of all intervention participants."

17b) For binary outcomes, presentation of both absolute and relative effect sizes is recommended

Does your paper address CONSORT subitem 17b? *

Copy and paste relevant sections from the manuscript (include quotes in quotation marks "like this" to indicate direct quotes from your manuscript), or elaborate on this item by providing additional information not in the ms, or briefly explain why the item is not applicable/relevant for your study

N/A

18) Results of any other analyses performed, including subgroup analyses and adjusted analyses, distinguishing pre-specified from exploratory

Does your paper address CONSORT subitem 18? *

Copy and paste relevant sections from the manuscript (include quotes in quotation marks "like this" to indicate direct quotes from your manuscript), or elaborate on this item by providing additional information not in the ms, or briefly explain why the item is not applicable/relevant for your study

N/A

18-i) Subgroup analysis of comparing only users

A subgroup analysis of comparing only users is not uncommon in ehealth trials, but if done, it must be stressed that this is a self-selected sample and no longer an unbiased sample from a randomized trial (see 16-iii).

	1	2	3	4	5	
subitem not at all important	○	○	○	○	○	essential

Does your paper address subitem 18-i?

Copy and paste relevant sections from the manuscript (include quotes in quotation marks "like this" to indicate direct quotes from your manuscript), or elaborate on this item by providing additional information not in the ms, or briefly explain why the item is not applicable/relevant for your study

N/A

19) All important harms or unintended effects in each group

(for specific guidance see CONSORT for harms)

Does your paper address CONSORT subitem 19? *

Copy and paste relevant sections from the manuscript (include quotes in quotation marks "like this" to indicate direct quotes from your manuscript), or elaborate on this item by providing additional information not in the ms, or briefly explain why the item is not applicable/relevant for your study

N/A

19-i) Include privacy breaches, technical problems

Include privacy breaches, technical problems. This does not only include physical "harm" to participants, but also incidents such as perceived or real privacy breaches [1], technical problems, and other unexpected/unintended incidents. "Unintended effects" also includes unintended positive effects [2].

	1	2	3	4	5	
subitem not at all important	○	○	○	○	○	essential

Does your paper address subitem 19-i?

Copy and paste relevant sections from the manuscript (include quotes in quotation marks "like this" to indicate direct quotes from your manuscript), or elaborate on this item by providing additional information not in the ms, or briefly explain why the item is not applicable/relevant for your study

Your answer

19-ii) Include qualitative feedback from participants or observations from staff/researchers

Include qualitative feedback from participants or observations from staff/researchers, if available, on strengths and shortcomings of the application, especially if they point to unintended/unexpected effects or uses. This includes (if available) reasons for why people did or did not use the application as intended by the developers.

	1	2	3	4	5	
subitem not at all important	○	○	○	○	○	essential

Does your paper address subitem 19-ii?

Copy and paste relevant sections from the manuscript (include quotes in quotation marks "like this" to indicate direct quotes from your manuscript), or elaborate on this item by providing additional information not in the ms, or briefly explain why the item is not applicable/relevant for your study

Your answer

DISCUSSION

22) Interpretation consistent with results, balancing benefits and harms, and considering other relevant evidence

NPT: In addition, take into account the choice of the comparator, lack of or partial blinding, and unequal expertise of care providers or centers in each group

22-i) Restate study questions and summarize the answers suggested by the data, starting with primary outcomes and process outcomes (use)

Restate study questions and summarize the answers suggested by the data, starting with primary outcomes and process outcomes (use).

	1	2	3	4	5	
subitem not at all important	○	○	○	○	○	essential

Does your paper address subitem 22-i? *

Copy and paste relevant sections from the manuscript (include quotes in quotation marks "like this" to indicate direct quotes from your manuscript), or elaborate on this item by providing additional information not in the ms, or briefly explain why the item is not applicable/relevant for your study

Not fully applicable - protocol paper.

"In this report, we describe the rationale, design, and initial implementation outcomes of a 3-arm pragmatic trial that assessed the feasibility and preliminary effectiveness of an eCDSS to improve CKD management in primary care compared with usual care. This study builds on previous pragmatic trials focused on EHR-based interventions to improve CKD management [28,29,51,52] and supports the feasibility of using EHRs to identify study participants, intervene in early CKD management, and measure study outcomes. Given its design as a pragmatic trial (per Pragmatic Explanatory Continuum Indicator Summary, or PRECIS, criteria [53]), we found high rates of participation by providers and low opt-out rates by patients. We also found high rates of eCDSS use by providers."

22-ii) Highlight unanswered new questions, suggest future research

Highlight unanswered new questions, suggest future research.

	1	2	3	4	5	
subitem not at all important	○	○	○	○	○	essential

Does your paper address subitem 22-ii?

Copy and paste relevant sections from the manuscript (include quotes in quotation marks "like this" to indicate direct quotes from your manuscript), or elaborate on this item by providing additional information not in the ms, or briefly explain why the item is not applicable/relevant for your study

Your answer

20) Trial limitations, addressing sources of potential bias, imprecision, and, if relevant, multiplicity of analyses

20-i) Typical limitations in ehealth trials

Typical limitations in ehealth trials: Participants in ehealth trials are rarely blinded. Ehealth trials often look at a multiplicity of outcomes, increasing risk for a Type I error. Discuss biases due to non-use of the intervention/usability issues, biases through informed consent procedures, unexpected events.

	1	2	3	4	5	
subitem not at all important	○	○	○	○	○	essential

Does your paper address subitem 20-i? *

Copy and paste relevant sections from the manuscript (include quotes in quotation marks "like this" to indicate direct quotes from your manuscript), or elaborate on this item by providing additional information not in the ms, or briefly explain why the item is not applicable/relevant for your study

"As a pilot study, this study was limited by its inclusion of patients and providers from a single institution. Therefore, both the intervention and strategies to encourage intervention uptake were adapted to meet the needs of this single institution that uses Epic Systems' EHR. However, we have found that the patient population in this clinic is similar to others [60], and Epic is a widely used EHR in multiple health systems. As a pragmatic trial, our sample size was smaller than initially anticipated and will thus ultimately impact our power to detect changes in our primary outcome. The short follow-up period for this pilot study will also limit our ability to assess some clinical outcomes. However, we were still able to use this pilot study to determine the feasibility of this intervention."

21) Generalisability (external validity, applicability) of the trial findings

NPT: External validity of the trial findings according to the intervention, comparators, patients, and care providers or centers involved in the trial

21-i) Generalizability to other populations

Generalizability to other populations: In particular, discuss generalizability to a general Internet population, outside of a RCT setting, and general patient population, including applicability of the study results for other organizations

	1	2	3	4	5	
subitem not at all important	○	○	○	○	○	essential

Does your paper address subitem 21-i?

Copy and paste relevant sections from the manuscript (include quotes in quotation marks "like this" to indicate direct quotes from your manuscript), or elaborate on this item by providing additional information not in the ms, or briefly explain why the item is not applicable/relevant for your study

"As a pilot study, this study was limited by its inclusion of patients and providers from a single institution. Therefore, both the intervention and strategies to encourage intervention uptake were adapted to meet the needs of this single institution that uses Epic Systems' EHR. However, we have found that the patient population in this clinic is similar to others [60], and Epic is a widely used EHR in multiple health systems. As a pragmatic trial, our sample size was smaller than initially anticipated and will thus ultimately impact our power to detect changes in our primary outcome. The short follow-up period for this pilot study will also limit our ability to assess some clinical outcomes. However, we were still able to use this pilot study to determine the feasibility of this intervention."

21-ii) Discuss if there were elements in the RCT that would be different in a routine application setting

Discuss if there were elements in the RCT that would be different in a routine application setting (e.g., prompts/reminders, more human involvement, training sessions or other co-interventions) and what impact the omission of these elements could have on use, adoption, or outcomes if the intervention is applied outside of a RCT setting.

	1	2	3	4	5	
subitem not at all important	○	○	○	○	○	essential

Does your paper address subitem 21-ii?

Copy and paste relevant sections from the manuscript (include quotes in quotation marks "like this" to indicate direct quotes from your manuscript), or elaborate on this item by providing additional information not in the ms, or briefly explain why the item is not applicable/relevant for your study

N/A

OTHER INFORMATION

23) Registration number and name of trial registry

Does your paper address CONSORT subitem 23? *

Copy and paste relevant sections from the manuscript (include quotes in quotation marks "like this" to indicate direct quotes from your manuscript), or elaborate on this item by providing additional information not in the ms, or briefly explain why the item is not applicable/relevant for your study

"Trial Registration: ClinicalTrials.gov NCT02925962;
https://clinicaltrials.gov/ct2/show/NCT02925962 (Archived by WebCite
at http://www.webcitation.org/78qpx1mjR)"

24) Where the full trial protocol can be accessed, if available

Does your paper address CONSORT subitem 24? *

Cite a Multimedia Appendix, other reference, or copy and paste relevant sections from the manuscript (include quotes in quotation marks "like this" to indicate direct quotes from your manuscript), or elaborate on this item by providing additional information not in the ms, or briefly explain why the item is not applicable/relevant for your study

This is a protocol paper. Full protocol can be accessed by contacting the author team.

25) Sources of funding and other support (such as supply of drugs), role of funders

Does your paper address CONSORT subitem 25? *

Copy and paste relevant sections from the manuscript (include quotes in quotation marks "like this" to indicate direct quotes from your manuscript), or elaborate on this item by providing additional information not in the ms, or briefly explain why the item is not applicable/relevant for your study

"LK and CP received funding from the National Institute of Diabetes and Digestive and Kidney Diseases (1R18DK110959). EK
received funding through the National Research Service Award (T32HP19025). The funders played no role in review or approval
of this manuscript for publication."

X27) Conflicts of Interest (not a CONSORT item)

X27-i) State the relation of the study team towards the system being evaluated

In addition to the usual declaration of interests (financial or otherwise), also state the relation of the study team towards the system being evaluated, i.e., state if the authors/evaluators are distinct from or identical with the developers/sponsors of the intervention.

	1	2	3	4	5	
subitem not at all important	○	○	○	○	○	essential

Does your paper address subitem X27-i?

Copy and paste relevant sections from the manuscript (include quotes in quotation marks "like this" to indicate direct quotes from your manuscript), or elaborate on this item by providing additional information not in the ms, or briefly explain why the item is not applicable/relevant for your study

This is an internal eCDSS was developed by the authorship team.

About the CONSORT EHEALTH checklist

As a result of using this checklist, did you make changes in your manuscript? *

- ○ yes, major changes
- ○ yes, minor changes
- ◉ no

What were the most important changes you made as a result of using this checklist?

Your answer

How much time did you spend on going through the checklist INCLUDING making changes in your manuscript *

1 hour

As a result of using this checklist, do you think your manuscript has improved? *

- ○ yes
- ◉ no
- ○ Other:

Would you like to become involved in the CONSORT EHEALTH group?

This would involve for example becoming involved in participating in a workshop and writing an "Explanation and Elaboration" document

- ○ yes
- ◉ no
- ○ Other:

Any other comments or questions on CONSORT EHEALTH

Your answer

STOP - Save this form as PDF before you click submit

To generate a record that you filled in this form, we recommend to generate a PDF of this page (on a Mac, simply select "print" and then select "print as PDF") before you submit it.

When you submit your (revised) paper to JMIR, please upload the PDF as supplementary file.

Don't worry if some text in the textboxes is cut off, as we still have the complete information in our database. Thank you!

Final step: Click submit !

Click submit so we have your answers in our database!

SUBMIT

Never submit passwords through Google Forms.

This content is neither created nor endorsed by Google. Report Abuse - Terms of Service

Google Forms
